# Supplementary material for: Experiences and preferences for psychosocial support: a qualitative study exploring the views of patients with chronic haematological cancers
Source: BMJ Open. 2023 Aug 18;13(8):e070467. doi: 10.1136/bmjopen-2022-070467 (PMC10441118; doi:10.1136/bmjopen-2022-070467)
Supplement: Supplementary data [file bmjopen-2022-070467supp001.pdf]

Supplementary file 1
Details of sample

ID	Diagnosis[1]	Age range at interview (years)	Years since diagnosis	Known treatment line(s) preceeding interview[2,3]					
				1	2	3	4	5	6
P1	CLL	60-70	4	Observation	-	-	-	-	-
P2	MZL	60-70	15	Observation	Chemotx	Observation	-	-	-
P3	CLL	60-70	22	Observation	Chemotx	Observation	-	-	-
P4	MZL	60-70	3	Observation	Chemotx	-	-	-	-
P5	MZL	50-60	2	HPE	Observation	-	-	-	-
P6*	CLL	70-80	8	Observation	Chemotx	Observation	-	-	-
P7*	CLL	60-70	6	Observation	Chemotx	Observation	-	-	-
P8	FL	70-80	3	Chemotx	Radiotx	Observation	-	-	-
P9	CLL	80-90	5	Observation	Chemotx	-	-	-	-
P10	FL	70-80	8	Observation	Chemotx	Chemotx	Chemotx	-	-
P11	Myeloma	60-70	10	Observation	Chemotx	Observation	-	-	-
P12	MZL	70-80	5	Observation	Chemotx	-	-	-	-
P13	CLL	50-60	1	Observation	-	-	-	-	-
P14	Myeloma	60-70	4	Steroids	Radiotx	Chemotx	Chemotx	Chemotx	SCT
P15	FL	70-80	3	Observation	Chemotx	-	-	-	-
P16	Myeloma	60-70	2	Chemotx	Chemotx	Chemotx	SCT	Observation	-
P17*	FL	60-70	3	Observation	-	-	-	-	-
P18	Myeloma	60-70	3	Chemotx	Chemotx	Chemotx	SCT	Observation	
P19	FL	50-60	3	Steroids	Chemotx	Chemotx	Observation	-	-
P20*	CLL	70-80	4	Observation	-	-	-	-	-
P21*	Myeloma	70-80	3	Steroids	Chemotx	Chemotx	Chemotx	SCT	-
P22*	CLL	70-80	3	Observation	Clinical trial	Observation	-	-	-
P23	Myeloma	60-70	3	Observation	-	-	-	-	-
P24	FL	50-60	4	Steroids	Chemotx	Radiotx	Observation	-	-
P25	FL	60-70	4	Chemotx	Chemotx	-	-	-	-
P26	Myeloma	70-80	4	Observation	-	-	-	-	-
P27*	CLL	70-80	4	Chemotx	Observation	-	-	-	-
P28	Myeloma	60-70	4	Steroids	Chemotx	Chemotx	SCT	Clinical trial	Chemotx
P29	CLL	70-80	3	Clinical trial	Observation	-	-	-	-
P30*	Myeloma	70-80	2	Observation	-	-	-	-	-
P31*	Myeloma	70-80	2	Radiotx	Steroids	Chemotx	Observation	-	-
P32*	MZL	60-70	2	Observation	Chemotx	Observation	-	-	-
P33	Myeloma	50-60	3	Chemotx	Chemotx	SCH	Observation	-	-
P34	FL	50-60	4	Steroids	Chemotx	Chemotx	Chemotx	-	-
P35	Myeloma	50-60	2	Chemotx	Chemotx	Chemotx	Chemotx	SCT	Observation

[1] CLL – Chronic Lymphocytic Leukaemia; FL – Follicular Lymphoma; MZL – Systemic Marginal Zone Lymphoma.
[2] Chemotx = Chemotherapy; HPE = H. Pylori eradication; Radiotx = Radiotherapy; SCT = Stem cell transplant (all autografts); SCH = Stem cell harvest (shown as SCT did not take place).
[3] Does not include supportive care (e.g. blood product transfusions, plasma exchange, bisphosphonates, cell mobilization products)
*Relative present at interview
