# Supplementary material for: Experiences and preferences for psychosocial support: a qualitative study exploring the views of patients with chronic haematological cancers
Source: BMJ Open. 2023 Aug 18;13(8):e070467. doi: 10.1136/bmjopen-2022-070467 (PMC10441118; doi:10.1136/bmjopen-2022-070467)
Supplement: Supplementary data [file bmjopen-2022-070467supp002.pdf]

Supplementary file 2

Topic guide

(Tested with two patients prior to use in interviews)

Patients and relatives were asked to focus on key time-points (diagnosis, W&W, treatment), with respect to the topics included in the following three areas:

1) Understanding and impact of chronic blood cancer

- Looking back, what did you know about blood cancers, and your diagnosis specifically?
- What led you to your diagnosis initially? *(symptoms, suspected cancer?)*
- How important is it to you that you receive information about your cancer? *(why is that?)*
- How do you feel about the information given to you at diagnosis/start of treatment?
- What did you understand about your diagnosis? *(and others?)*
- What were your expectations of what would happen after diagnosis? *(treatment, follow-up)*
- What issues did you discuss at the time of your diagnosis? *(disease type, likely pathway, outcomes?)*
- What did you understand about your likely pathway and outcomes? *(immediate and later?)*
- What do you understand about your treatment?
- How do you feel about the future? *(optimistic, pessimistic?)*
- What impact has having a chronic blood cancer had on you? (activities, work, psychosocial issues)
- What strategies have you used to help you cope with your blood cancer?

2) Information

- How do you feel about getting information from HCPs generally? *(time constraints; use of language/terminology)*
- Do you feel the information given applies specifically to you? *(personalized, tailored, specific)*
- How healthcare practitioners (HCPs) ascertain your information needs?
- Is the information you received explained in a way you can understand? *(technical language; level of detail)*
- What do HCPs do to check if you understand the information they give you?
- How do you feel about asking questions? Are your questions always answered?
- Do you feel that your information needs are usually met? What worked well and could have been better? *(diagnosis; treatment initiation/cessation - examples)*
- What do you think about the timing of information from HCPs? When is the right time? *(at diagnosis; during clinic appointments; when disease status changes; at other times)*
- How do/did you feel about discussing the risks/benefits of different treatments with HCPs?
- How do you feel about discussing prognosis? *("a statement about expectations that refers to the likely course of the cancer and/or outcome") (want to know/not; timing; language)*
- What strategies do you use to absorb information? (*in general, how bad news is processed*)

3) Treatment decisions

- How do you feel about being involved in decisions with HCPs about your treatment?
- Have you been asked you if you want to be involved in decisions about treatment?
- Do you want to be involved in decisions? *(preference for patient only; clinician only; patient/clinician)*
- What should be considered during treatment decision making? *(effectiveness of treatment; side effects; prognosis; patient goals, values, preferences; impact on quality of life)*
- What might make it easier or harder for you to be involved in making decisions about your treatment? *(time; style of communication; how information is conveyed; explanations)*
- Are there particular time-points when it is harder to be involved in making decisions about treatment? *(diagnosis; treatment initiation/change; treatment cessation)*
