# Supplementary material for: Experiences and preferences for psychosocial support: a qualitative study exploring the views of patients with chronic haematological cancers
Source: BMJ Open. 2023 Aug 18;13(8):e070467. doi: 10.1136/bmjopen-2022-070467 (PMC10441118; doi:10.1136/bmjopen-2022-070467)
Supplement: Author's manuscript [file bmjopen-2022-070467.draft_revisions.pdf]

BMJ Open is committed to open peer review. As part of this commitment we make the peer review history of every article we publish publicly available.

When an article is published we post the peer reviewers' comments and the authors' responses online. We also post the versions of the paper that were used during peer review. These are the versions that the peer review comments apply to.

The versions of the paper that follow are the versions that were submitted during the peer review process. They are not the versions of record or the final published versions. They should not be cited or distributed as the published version of this manuscript.

BMJ Open is an open access journal and the full, final, typeset and author-corrected version of record of the manuscript is available on our site with no access controls, subscription charges or pay-per-view fees (http://bmjopen.bmj.com).

If you have any questions on BMJ Open's open peer review process please email info.bmjopen@bmj.com

BMJ Open

Experiences and preferences for psychosocial support: A qualitative study exploring the views of patients with chronic haematological cancers

Journal:	*BMJ Open*
Manuscript ID	bmjopen-2022-070467
Article Type:	Original research
Date Submitted by the Author:	24-Nov-2022

Complete List of Authors:	Sheridan, Rebecca; University of York, Health Sciences<br>McCaughan, Dorothy; University of York, Health Sciences<br>Hewison, Ann; University of York, Health Sciences<br>Roman, Eve; University of York, Health Sciences<br>Smith, Alexandra; University of York, Health Sciences<br>Patmore, Russell; Castle Hill Hospital<br>Howell, Debra; University of York, Health Sciences

Keywords:	Leukaemia < HAEMATOLOGY, Lymphoma < HAEMATOLOGY, Myeloma < HAEMATOLOGY

SCHOLARONE™
Manuscripts

I, the Submitting Author has the right to grant and does grant on behalf of all authors of the Work (as defined in the below author licence), an exclusive licence and/or a non-exclusive licence for contributions from authors who are: i) UK Crown employees; ii) where BMJ has agreed a CC-BY licence shall apply, and/or iii) in accordance with the terms applicable for US Federal Government officers or employees acting as part of their official duties; on a worldwide, perpetual, irrevocable, royalty-free basis to BMJ Publishing Group Ltd ("BMJ") its licensees and where the relevant Journal is co-owned by BMJ to the co-owners of the Journal, to publish the Work in this journal and any other BMJ products and to exploit all rights, as set out in our licence.

The Submitting Author accepts and understands that any supply made under these terms is made by BMJ to the Submitting Author unless you are acting as an employee on behalf of your employer or a postgraduate student of an affiliated institution which is paying any applicable article publishing charge ("APC") for Open Access articles. Where the Submitting Author wishes to make the Work available on an Open Access basis (and intends to pay the relevant APC), the terms of reuse of such Open Access shall be governed by a Creative Commons licence – details of these licences and which Creative Commons licence will apply to this Work are set out in our licence referred to above.

Other than as permitted in any relevant BMJ Author's Self Archiving Policies, I confirm this Work has not been accepted for publication elsewhere, is not being considered for publication elsewhere and does not duplicate material already published. I confirm all authors consent to publication of this Work and authorise the granting of this licence.

For peer review only

Experiences and preferences for psychosocial support: A qualitative study exploring the views of patients with chronic haematological cancers

AUTHORS: Rebecca Sheridan, Dorothy McCaughan, Ann Hewison, Eve Roman, Alexandra Smith, Russell Patmore, Debra Howell

CORRESPONDING AUTHOR:

Debra Howell
Epidemiology & Cancer Statistics Group, Department of Health Sciences, Seebohm Rowntree Building, University of York, York, UK. YO10 5DD.
Telephone: +44 (0)904 321895
Email: debra.howell@york.ac.uk

CO-AUTHORS

Rebecca Sheridan
Epidemiology & Cancer Statistics Group, Department of Health Sciences, Seebohm Rowntree Building, University of York, York, UK. YO10 5DD.
Telephone: +44 (0)904 321079
Email: rebecca.sheridan@york.ac.uk

Dorothy McCaughan
Epidemiology & Cancer Statistics Group, Department of Health Sciences, Seebohm Rowntree Building, University of York, York, UK. YO10 5DD.
Telephone: +44 (0) 904 321348
Email: dorothy.mccaughan@york.ac.uk

Ann Hewison
Epidemiology & Cancer Statistics Group, Department of Health Sciences, Seebohm Rowntree Building, University of York, York, UK. YO10 5DD.
Telephone: +44 (0) 904 321916
Email: ann.hewison@york.ac.uk

Eve Roman
Epidemiology & Cancer Statistics Group, Department of Health Sciences, Seebohm Rowntree Building, University of York, York, UK. YO10 5DD.
Telephone: +44 (0) 904 321882
Email: eve.roman@york.ac.uk

Alexandra Smith
Epidemiology & Cancer Statistics Group, Department of Health Sciences, Seebohm Rowntree Building, University of York, York, UK. YO10 5DD.
Telephone: +44 (0) 904 321884

For peer review only

For peer review only - http://bmjopen.bmj.com/site/about/guidelines.xhtml

Email: alexandra.smith@york.ac.uk

Russell Patmore
Queens Centre for Oncology and Haematology, Castle Hill Hospital, Cottingham, UK.
Telephone: +44 (0) 482 604444
Email: russell.patmore@nhs.net

KEYWORDS: Haematological cancer; psychosocial; peer support; multiple myeloma; leukaemia; lymphoma

WORD COUNT: 5662, including all quotations

For peer review only

ABSTRACT

Objectives Patients with chronic haematological cancers are often treated on a relapsing-remitting pathway, which may extend for many years, meaning they are susceptible to prolonged emotional distress. Patients experiences and preferences with regards to psychosocial support are relatively unexplored, which our study aimed to address.

Setting and design A qualitative, exploratory study, using semi-structured interviews, set within the UK's Haematological Malignancy Research Network (HMRN: www.HMRN.org), an ongoing population-based cohort which aims to improve clinical practice.

Participants Thirty-five patients with one of the following diagnoses: chronic lymphocytic leukaemia, follicular lymphoma, marginal zone lymphoma, and myeloma; 10 were interviewed alongside a relative.

Results Six themes were identified: (i) accessing support; (ii) individual coping behaviour affecting support preferences; (iii) divergent and fluctuating thoughts on patient support forums; (iv) the role and influence of family and friends; (v) other sources of support and outstanding needs; (vi) the importance of support for family and carers. Our findings suggest that patients' individual attitudes towards support varied over time. This also influenced whether support was perceived to be available, and if it was then utilised by patients.

Conclusion This study highlighted the variation in preferences towards psychosocial support for patients with chronic haematological cancers. As patients may live with the significant emotional impact from their cancer for many years, psychosocial wellbeing should be monitored frequently, with holistic support provided where needed.

ARTICLE SUMMARY

Strengths and limitations of this study

- The number of interviews (35) allowed for the inclusion of patients diagnosed with a range of chronic hematological cancers.
- Purposive sampling enabled us to interview patients with varying demographics and treatment pathways.
- Patient recall may be impacted by prolonged time since diagnosis, despite the use of diaries and calendars during interviews.
- Relatives were not present at every interview, which may limit findings with regard to views about support for carers and relatives.
- Our findings are likely to be transferable within the UK, and in countries with similar approaches to healthcare, though we acknowledge they are unlikely to be generalizable to the whole population.

For peer review only

For peer review only - http://bmjopen.bmj.com/site/about/guidelines.xhtml

INTRODUCTION

Haematological cancers are a diverse group of disorders, with a number of sub-types (1). Broadly classified as leukaemias, lymphomas and myeloma, collectively they are the fifth most common cancer globally (2), with an increasing incidence amongst ageing populations (3,4). While some of these cancers (such as diffuse large B-cell lymphoma) are potentially curable, the majority (around 60%) are not; with treatments aiming to ameliorate symptoms and slow down progression (5). The four commonest chronic haematological cancers (CHCs) are chronic lymphocytic leukaemia (CLL), myeloma, follicular lymphomas (FL), and marginal zone lymphoma (MZL). Some patients with such conditions may be treated from diagnosis; others do not need immediate treatment and will be managed on 'watch and wait', either never requiring treatment, or alternating between treatment and observation with the aim of restoring remission (6). Treatments may include combinations of chemotherapy, radiotherapy, stem cell transplant and targeted agents (7–9).

Patients with CHCs may be successfully managed on remitting-relapsing pathways over many years, though symptom burden and loss of functional ability can cause increased dependence on others, and have a detrimental effect on quality of life (10–14). Living with a CHC may be associated with increased emotional and psychological distress, linked primarily to uncertainty concerning disease progression/relapse, treatment, and prognosis, compounded by poor diagnostic understanding, and informational deficiencies (5,15–24). The importance of these patients receiving timely psychosocial support during observation, and before, during, and after intensive treatments is underscored in El-Jawahri et al.'s (2016) prospective, longitudinal study, showing patients at increased risk of developing psychological disorders when supportive needs are not met (25).

CHC patients' experiences of accessing and receiving support remains relatively unexplored, despite the growing significance of this issue due to improved outcomes; five-year relative survival estimates range from 48% (myeloma), to 88% (FL) (26). Relevant literature emphasises the value of family and significant others to enable patient adjustment and coping (15,20,27,28). Patients may also rely on peer support groups and health care professionals (HCPs), such as clinical nurse specialists (CNSs), for emotional as well as informational support (20,28).

Understanding patients' views of seeking/receiving support, and associated barriers and facilitators, is circumscribed by the small-scale of many published studies, predominantly focusing on the perspectives of myeloma patients (15,16,21,23,27). Our own interview study therefore elicited in-depth perspectives from a comparatively large qualitative sample (35) of patients with CLL, FL, MZL and myeloma. Data were explored with the aim of examining the experiences of seeking/receiving emotional and psychological support, from the point of diagnosis onwards, and identifying perceived needs and preferences.

METHODS

This study is part of a broader project examining the perspectives of CHC patients concerning information needs and treatment decisions. Further details can be found elsewhere (5). Methods and results are reported according to the Consolidated Criteria for Reporting Qualitative Research guidelines (29).

For peer review only - http://bmjopen.bmj.com/site/about/guidelines.xhtml

Study design and setting

A qualitative, exploratory study, using semi-structured interviews, set within the UK's Haematological Malignancy Research Network (HMRN) (30). HMRN's population-based patient cohort was established in 2004, incorporating around 4 million people with similar socio-demographics to the UK. HMRN is an on-going collaboration between university academics, National Health Service (NHS) clinicians, and patients and carers, who co-design and conduct research, with the aim of improving clinical practice, locally, nationally and internationally.

Sampling

Sampling was purposive; we aimed to capture a range of experiences relating to the topic of interest (31). Participants with CLL, FL, MZL or myeloma were selected according to the median diagnostic age for each cancer, with variation by gender, time since diagnosis, and time points on the clinical pathway. Refinement of the sampling strategy led to inclusion of patients across broader demographic categories; for example, by including those above and below sub-type median diagnostic age.

Data collection

Checks with patients' clinical teams ensured they were well enough to participate in the study. Identified individuals were sent information and the researcher's contact details to discuss participation. Interviews were conducted by an experienced, female researcher (DM), between February-October 2019, at a time and place of the patient's choosing, usually their home, with a friend or relative present if desired. Participants were invited to ask questions about the study prior to interview, and assurances were given regarding anonymity and confidentiality. Written consent was obtained from all participants, including for the use of direct quotations. Interviews lasted 60-90 minutes, and were digitally audio-recorded. A semi-structured topic guide (Supplementary file 1), developed from the research literature, with haematology practitioners, was used flexibly to guide discussions, which were subsequently transcribed verbatim, and checked, corrected and anonymised by the interviewer.

Data analysis

Reflexive thematic analysis was used, an approach more akin to a transtheoretical technique, than a methodology predicated on a particular theoretical stance (32,33). Analysis was carried out by an experienced research team (RS, DM, DH, AH), using an exploratory, inductive approach, underpinned by the research questions. Coding and development of themes, based on active searching for patterns of meaning across the data set, was a collaborative process, achieved through the dual processes of immersion in the data, and distancing, to allow time for reflection and development of insights. Regular team meetings focussing on theme review and refinement, supplemented by written memos and reflections, promoted critical thinking, and enhanced reflexivity and interpretive depth. The overall aim was to 'interrogate' the data to arrive at an interpretation that was *'insightful, thoughtful, rich…nuanced'* (33). Patient quotes are written in italics and refer to participant numbers (e.g. P1 for the patient and P1R for the patient's relative).

Patient and public involvement

Patients and relatives were involved in prioritising research aims, preparing the funding application and disseminating findings.

For peer review only - http://bmjopen.bmj.com/site/about/guidelines.xhtml

RESULTS

Thirty-five patients participated in the study; ten relatives contributed to interviews. Twelve patients had myeloma, 10 CLL, 8 FL and 5 MZL; treatment pathways varied. The majority (22) were aged ≥60 years; and 19 were male. Further details can be found in Supplementary file 2.

Six themes were identified: (i) accessing support; (ii) individual coping behaviour affecting support preferences; (iii) divergent and fluctuating thoughts on patient support forums; (iv) the role and influence of family and friends; (v) other sources of support and outstanding needs; (vi) the importance of support for family and carers. Individual preferences were evident in each theme, with the patient's personality impacting their use of support (Figure 1).

Theme 1: Accessing support

Subtheme 1.1 Varied types of support

Patients reported accessing or awareness of various types of peer support, including face-to-face/online forums, and being introduced to, or knowing, other patients who shared their diagnosis. The majority of face-to-face support groups were not disease specific, but were for those with any haematological cancer. Online cancer specific groups referenced included Myeloma UK, CLL Support Association and also Facebook groups.

Informal support from family and friends was particularly important, whilst nurses, and, sometimes doctors, also provided support. Patients utilised NHS and private counselling services and complementary therapies such as acupuncture. Macmillan centres were attended by some, as well as support days and events hosted by charities, such as Myeloma UK.

Subtheme 1.2 Knowing where to find support

Most patients knew about online and face-to-face support groups, even if they did not want to use these resources. It was not always clear how patients became aware of support groups; some were told by their doctor or nurse (P10, P12, P28), or received information via leaflets (P5). P19 and P3 specifically noted finding American websites/forums; P19 found *'it wasn't anything particularly helpful'* due to their American focus.

Some patients contacted others who shared their diagnosis via their GP or family and friends. For example, P14's GP organised for someone with the same condition to speak to him: *'hearing from somebody who's been through it all and got the badge certainly helped'*. P33 had requested a *'buddy'*, something P21R would have welcomed for herself and her relative: *'someone that's like a year in to having this, who would sit down and explain what they discovered and how it works, and what to expect'*.

Some patients (P1, P4) requested counselling, while others (P9, P13) were offered a referral by nursing staff. Nurses also informed some patients (P8, P25) about other forms of support such as therapeutic massage. P16 had not been told about Macmillan support centres, but would have found this useful; it was unclear whether other patients were informed about them.

Subtheme 1.3 Impact of disease subtype on accessing peer support

A number of patients commented on difficulties due to their cancer subtype, such as its rarity (P5, P25, P33). Because of this, P25 felt *'on me [sic] own'* and unsupported, noting that neither her nor

For peer review only - http://bmjopen.bmj.com/site/about/guidelines.xhtml

her daughter could find any support groups. This could be problematic even for patients who shared the same diagnosis; P5 explained people at her support group all had lymphoma or leukaemia but she didn't perceive herself as having the same disease due to differences in site, clarifying: *'I have [the same cancer] but it's in my stomach'.*

Theme 2: Individual coping behaviour affecting support preferences

The patient's personal approach to their cancer influenced their coping strategies, often resulting in a reluctance to access formal support.

Subtheme 2.1 Perceptions of illness: Distancing, distraction, fighting back and maintaining hope

A number of patients didn't want to '*dwell'* on their cancer and discussed measures taken to actively distance or distract themselves. Instead of seeking specific support, such individuals focused on other activities, with P17 saying: *'We have quite a lot of interests, so we're always doing things and I think that's good because it just takes your mind off things'.* P27 and P29 took a philosophical approach to their cancer: *'there's nothing you can do about it…you make the best…of what is available for you'* (P27). Patients did not want to be defined by their condition, or for their condition to *'rule [their] life'* (P3). P5 attended a support group infrequently, as they didn't want their cancer to '*represent'* them. Similarly, P35 commented: *'for some people [at support group] it's like their whole life is having myeloma. I just don't want to be like that'.*

Many patients highlighted specific coping strategies to distract themselves from their CHC, including exercising, dog walking and gardening. Having another focus was important to P3 when trying to accept their diagnosis and deferred treatment (watch and wait): *'[running] made a massive difference because I'm doing something…I'm fighting back'*.

Some people felt they needed to stay positive and maintain hope with respect to their cancer and future outcomes. P24 adopted a '*positive mental attitude'; P25* described focussing on future events as '*summit to aim for'.* P13 felt that support group attendance might interfere with their positive approach due to the sharing of negative experiences, explaining: *'I wouldn't want to hear all of the things, you know, "well, it did this to me and it did that to me, and I thought I was on death's door", …I need to keep buoyant and positive about it'.*

Subtheme 2.2 Perceptions of personality

Some patients felt they were not the 'type' to attend a support group: *'I am just a bit introverted meself [sic]…I'm not a kind of a support group type person…'* (P21). Similarly, P27 described support groups as *'not for me'*, but did acknowledge that this could change in the future. Whilst P10 appreciated that support groups could be useful for others, they found them unnecessary due to them being inherently positive: '*I have a positive attitude and I don't feel I need support'.*

Theme 3: Divergent and fluctuating thoughts on patient support forums

Patients referred to both the significant benefits and also drawbacks of face-to-face and online support forums, with variations noted between individuals.

For peer review only - http://bmjopen.bmj.com/site/about/guidelines.xhtml

Subtheme 3.1 Varied perspectives

From a positive perspective, support groups were described as *'very valuable'* (P1); P6 described their group leader as their '*rock'.* Meeting others in a similar situation provided patients with reassurance and comradery: *'you're not on your own but to know somebody else is going through it, and you can tell her your worries'* (P3). P33 also noted that their efforts to maintain positivity within support groups had helped others, saying that members had remarked: *'you've cheered me up and made me feel positive because you're always so positive'.*

Whilst positive experiences were welcomed, hearing negative accounts could be frightening, reflecting patients' desires not to be exposed to these stories (see subtheme 2.1). For example, P35 explained: '*I thought I'd find [support group] terrifying, which I sort of do really because it's like people just talking about their terrible stories a lot of the time'.* P7 highlighted that patients may hear both positive and negative accounts, with the negative making them feel like they were '*in a queue sat there waiting to die'.*

Subtheme 3.2 Changing preferences over time

Patients commented on their decisions regarding when to seek group support, often depending on the status of their cancer. For example, some patients (P4, P8) did not attend face-to-face support groups during treatment due to fatigue, illness or infection risk. Engagement could increase when they needed information or advice, such as when making treatment decisions. As explained by P3, who became more active on internet forums at relapse: *'A lot of people were saying, yeah I was the same as you…I just wanted to forget about it but then when you have something to remind you that you've still got the condition a lot of people start to be more active'*. Some patients on observation didn't feel a support group was necessary as they were '*coping well'* (P17), or because their cancer didn't '*affect [their] lifestyle'* (P20).

Subtheme 3.3 Information provision

Patients generally found support groups and websites a useful source of information, and some noted that they themselves had provided information to others (P6, P13, P19). On websites in particular, patients could choose what information they wanted to engage with, and when. For example, P19 found negative material unwelcome saying: *'it used to scare me'*. P3 highlighted the importance of understanding that *'everyone is different', explaining: 'you have to decide what suits you, what you feel comfortable with and learn to take the advice that's useful for you and ignore the advice that's less useful'.*

Theme 4: The role and influence of family and friends

Subtheme 4.1 Emotional and practical support: Reassurance, companionship and the importance of family and friends

Family and friends provided significant emotional support to the majority of patients; for example, P2 commented: *'the people who are close to you are very important because they can keep you going…I think they're very important in reassuring you'*. Patients also acknowledged the importance of practical support, including relatives or friends accompanying them to hospital appointments, reminding them what was discussed, and learning about their condition. P9 explained '*my daughter knows as much about my treatment as I do. She keeps really up-to-date with it and she's with me*

For peer review only - http://bmjopen.bmj.com/site/about/guidelines.xhtml

most treatments'. Contrastingly, P3 attended appointments alone, because his wife would *'worry herself silly',* as appointments were *'more stress inducing'* for her than for himself. P34, who attended appointments alone due to his wife's work commitments, acknowledged that, with hindsight, practical support would have been useful.

Emotional and practical support from friends was particularly important for patients without immediate family support. P8, who lived alone, described her friend as '*marvellous',* explaining: *'I haven't got any family…She was just there for me the whole time'.* P33, whose partner had died, felt they needed practical and emotional support: *'I have to do all my own research, all my own ringing around and sometimes I think I just wish I had that person…I want somebody just to hold my hand and go, I'm going to sort that for you'.* For some, having family did not guarantee support, as exemplified by P25, who relied on a friend to provide emotional and practical support, despite living with relatives.

Subtheme 4.2 Disease characteristics and family dynamics: Impact of the cancer, perceived response of others and life context

Patients' decisions about disclosing their CHC impacted available support, and were often influenced by factors relating to their cancer. When patients felt their diagnosis would impact their lives, they often chose to tell family and friends. For example, P22 explained: *'we knew we were always going to always be at hospital…so we took the road that we would tell them'.* In contrast, when patients didn't feel their cancer was noticeably impacting them, for example when on observation, they did not always disclose their diagnosis, thus eliminating opportunities for support. P2 said: *'close family knew about it and close friends and that was it because I could get on with me [sic] life. I could manage it. Nobody needed to know'.* P20 also highlighted the nature of 'watch and wait', explaining they did not inform people about their diagnosis as: *'they wouldn't understand that there's no treatment required'.*

Patients hesitated to disclose their CHC to children for various reasons including their young age, because they were undertaking important exams, and/or because they did not wish to be '*a burden'* (P32). P35 knew she had to tell her children as her diagnosis was *'a big life changing event'* but was unsure about the timing, explaining: '*I just wanted to know if I could carry on my life without telling them'.* Being on watch and wait allowed P11 to keep their diagnosis from their children, who they believed were too young to understand/cope.

Even where family and friends were informed about the diagnosis, feelings were not always shared. For example, P7R explained that they did not discuss their worries in order to '*protect'* family. Similarly, P5 felt they had to be strong for others, so were providing, rather than receiving, support. Some patients did not share their feelings about their CHC even with close family, or were reluctant to ask for help, so could not be supported. For example, P2 described shock at their diagnosis, but his wife had said: *'the trouble was you didn't tell me all this so I couldn't be involved'.* P25 described feeling lonely, but wanted to *'make it easier for them [family]'*, admitting: *'I won't ask nobody for help. If somebody, if me [sic] cousin had offered to come, I would have said, "no I'm fine"'.*

Family dynamics could change as a result of the CHC diagnosis. P19 said: *'I didn't feel well and for the first time ever, my family just took full control of everything and normally I'm the one that's like the matriarch'.* However, whilst the patient was grateful, the diagnosis and changing circumstances were not accepted by everyone: *'[Daughter] wouldn't even talk about it and to this day she doesn't really. She just, you know, she just likes her mum to be mum, and functional'.*

For peer review only - http://bmjopen.bmj.com/site/about/guidelines.xhtml

Theme 5: Other sources of support and outstanding needs

Subtheme 5.1 Perceptions of support from healthcare professionals

Nursing staff were considered a valuable source of support, often providing reassurance and encouragement for patients. For example, P14 said: '*[Nurse] has been there for me and guided me, cajoled me, pointed me in the right direction'*. There were differences in the type of support patients perceived nurses as providing; P5 described it as *'medical'* but '*not necessarily psychological'*, whilst P7 said: *'As well as your physical condition [nurses] were bothered about my emotional condition…'.*

A number of patients (P1, P16, P34) reported support from nurses as reliable and always available. In contrast, P4 explained: *'I didn't feel I could be ringing [nurse] every day…because obviously she's got lots of other things to do and lots of other people to deal with'*. P4 acknowledged most nurses were *'understanding'*, but felt some trivialised her concerns: '*you'd ring up and they'd sort of brush it off'.* P4 and P33 expressed disappointment when nurses reneged on their stated intentions, including referral for counselling, and providing reassurance to family.

Interactions with nursing staff appeared to vary according to CHC management, with patients on treatment having greater contact than those on watch and wait, and were also influenced by patient preferences; for example, P2 (prolonged watch and wait before treatment), described receiving contact details for nursing staff but choosing not to use them. Others on watch and wait appreciated the contact details and felt support was available if needed (P13, P17, P26). P3 (also on watch and wait prior to treatment) said: *'The only time I can remember talking to nurses a lot was when I had my treatment and of course they were absolutely superb'.* Similarly, a number of patients with myeloma (P11, P14, P18, P28), in frequent contact with nurses during treatment, discussed the importance of receiving regular support: '*we've had a lot of dealing with [nurses] and they're spot on'* (P11). Contrastingly, P13 (watch and wait since diagnosis) perceived nurses as focusing on providing '*holistics…mind, body and soul'* care to those on watch and wait, in order to '*free up the doctors, so they can see the patients that maybe need a little bit of extra time*'.

Whilst most comments about support from HCPs were about nurses, some patients referred to support from doctors, which was mainly, but not uniformly, perceived positively. P9 felt their doctor was *'very supportive and helpful'* when they were upset about needing chemotherapy, and P12 felt their doctor had '*plenty of empathy'.* In contrast, P4 described their doctors as speaking on a *'consultant level, with no emotions'.* A number of patients highlighted positive interactions with their GPs, with P30 describing theirs as *'fantastic'*. However, others (P6, P13, P23, P25) felt their GP lacked knowledge about their CHC. Three patients mentioned receiving support from other HCPs outwith the NHS, such as Myeloma UK nurses (P29) and Macmillan nurses (P13, P19).

Subtheme 5.2 Counselling and complementary support

Seven patients discussed their experiences of counselling, with perceptions varying markedly. P1 felt this had been useful, facilitating the realisation that they had '*experienced a tremendous trauma*', which had impacted their behaviour in response to treatment and infection fears. Conversely, P25 felt counselling was '*a complete waste of time'* and not what she had expected, explaining: *'To me, a counsellor is somebody that will counsel you and advise you. She never opened her mouth'.* Nevertheless, P25 did feel the service had helped her '*come to terms*' with her diagnosis. P33 felt their psychologist only cared how they felt about their treatment, and not their wellbeing more

For peer review only - http://bmjopen.bmj.com/site/about/guidelines.xhtml

generally. P35 described both positive and negative aspects of counselling, explaining that whilst it was '*helpful*' it could also be upsetting to discuss their feelings.

Four patients (P9, P16, P19, P22) referred to Macmillan services, all positively, though P22 felt these weren't aimed at her as she wasn't *'terminal'*. A number of patients discussed complementary medicines including acupuncture (P9), therapeutic massage (P8, P25) and reflexology (P23). P2 talked at length about the reassurance and support provided by his homeopath who he had attended for several years, comparing interactions to those with their doctor: *'I used to get different doctors and you just felt as though you were part of a process but…my homeopath I go to, we sit and we talk about my symptoms, how I'm feeling'.*

Subtheme 5.3 Unmet needs

Some patients highlighted unmet support needs. P4, who was waiting for counselling, noted: '*I didn't feel like there was anybody professional shall I say, who I could talk to and just tell them how I felt and that I was really scared...'*. P25 commented that: *'[leaflets] could do a little bit more on your thoughts. Yeah on your mind, on the emotional side of it',* whilst P5 felt they would cope with their CHC psychologically, but '*at a price'.*

A number of patients identified key points in the CHC trajectory which caused emotional turmoil. Many patients highlighted the *'shock'* of diagnosis, *'a huge emotional rollercoaster'* (P7), and identified a lack of emotional support at this critical juncture in their lives. P13 described becoming upset when she discovered '*lumps'* which could indicate disease progression; others described feeling shock at being told they would need treatment after a period of observation (P3, P15). Patients who had experienced 'watch and wait' alluded to the difficulty of living with uncertainty: '*it will flare up again...that causes anxiety…you don't know when…it's lurking there'* [P4].

Theme 6: The importance of support for family and carers

This was discussed by several patients, with P12 explaining they felt support was '*just as important for the family as it is for the patient'.* Patients appreciated that family are also impacted by their diagnosis; P11 suggested '*it's probably harder on my wife or my children'.* Similarly, P28 remarked that: '*The worry is done by the carers'.* In contrast, P6 acknowledged differences in perceptions of burden between the patient and their family: *'Mentally, [husband] forgets sometimes and he admits – "I forget you've got leukaemia". But it lives with me 24/7. I never forget that I've got it'.*

Patients' families found support in various settings. For example, P12 and P13 described nurses offering support to relatives, with P12 commenting that speaking to the nurse '*kind of settled [wife] a bit'.* P11 encountered another patient, also with myeloma, and explained how their wives then talked and found this useful: '*[Wife (of P11)] was a bit left in the dark she felt, so she really enjoyed talking to [contact's wife] about how she felt'.* Support was also available via Cancer Centres, with P18's partner accessing counselling and a therapeutic course. P28 praised Myeloma UK for their approach to supporting both patients and carers, including holding sessions specifically for carers at their events.

In contrast, some family members commented that their support needs were unmet. P7R described there being no information about support for relatives, saying '*it's lonely',* and highlighting that the carer also has a *'burden'.* P21R felt unsupported, suggesting counselling should be provided for patients and relatives. Sometimes support was available, but relatives were unwilling to accept it. For example, P19 commented that her daughter really needed support but wouldn't access it: *'the*

For peer review only - http://bmjopen.bmj.com/site/about/guidelines.xhtml

Macmillan nurse was really good and she offered to speak to [daughter], and she tried to ring her and she didn't want to talk about it'.

DISCUSSION

This study provides novel in-depth insights about the experiences of CHC patients in seeking, accessing and engaging with psychosocial support. Many patients relied on support groups, which were generally perceived positively, but there were also significant barriers to their use, including: distress on hearing negative stories, lack of relevant groups due to the rarity of the cancer, and others' poor outcomes interrupting patients' positive approaches to their illness. Family and friends provided psychosocial and practical support, though living with family did not guarantee support. Furthermore, individual patients' personality, outlook on life, and behaviour, impacted on their desire to seek and receive support, and on the family's ability to provide it, with some patients choosing not to disclose their diagnosis. Our study also indicated that positive interactions with HCPs, most notably nursing staff, appeared to be largely determined by the patient's pathway; the majority of those who considered nurses as emotionally supportive had received treatment. Finally, both patients and relatives discussed the impact of the CHC on family and friends, stressing their support needs and highlighting gaps in current provision.

The unique nature of CHCs likely impacts upon patients' experiences of psychosocial support. For example, patients who did not disclose their diagnosis tended to be those with the most indolent disease, who did not require treatment, and/or who felt their cancer was not impacting their life and was invisible to others. This lack of disclosure may not have been possible for patients with cancer types that are more symptomatic and/or require immediate and aggressive treatment. Accordingly, some patients highlighted reluctance to help family and friends understand why treatment was not currently necessary, which was likely to have contrasted with their expectations of the medical response following a cancer diagnosis. In addition, unlike many potentially curable cancers, relapse and further intervention is always possible, or in some subtypes, expected. Therefore, the emotional impact of living with a CHC is likely to be variable and fluctuating, particularly given patients increasing life expectancy. Ongoing support, which is responsive to the patient's position on their relapsing-remitting pathway, and their associated needs, is especially important.

Our findings are consistent with previous research observing barriers to support group attendance. For example, Swash et al (2018) describe patient difficulties in remaining positive when others with the same diagnosis become increasingly unwell, or die. A reliance on family and friends for support has also been reported (27,28,34). Other research discusses patients withholding anxiety and distress about their cancer from friends and relatives to protect them from worry (15). Generic (e.g. Macmillan) cancer support services were rarely mentioned by interviewees in our study and some deemed these inappropriate for CHC. This echoes previous research which found people with indolent blood cancers didn't always identify themselves as cancer patients, largely due to their prolonged pathway, lack of obvious symptoms, and associated terminology (e.g. haematology not oncology) (20). Limited HCP contact during observation has also been highlighted, with hospital appointments occurring at lengthy (e.g. 3-12 month) intervals, thus reducing opportunities for patients to receive emotional support (20,35). Furthermore, some patients do not feel comfortable raising emotional concerns with HCPs, and feel HCPs do not want to discuss these, thereby enhancing barriers to support, findings which resonate with those from our study (20).

The recent National Cancer Patient Experience Survey (NCPES) found 81.5% of patients, including those with haematological cancers, said they had a nurse to support them during treatment, with

For peer review only - http://bmjopen.bmj.com/site/about/guidelines.xhtml

most finding them reliable and helpful (36). HCPs are also in a position to recommend and increase awareness of sources of support (37), with the NCPES reporting that 89.6% of patients felt hospital staff provided guidance on support groups and other resources, and 75% indicating they received adequate support with their health and wellbeing. However, only 31.8% could access emotional support post-treatment (36), which is likely to be particularly relevant to those with CHCs. Whilst specialist nurses are said to appreciate the potential for CHC patients' emotions to change over time (38), further study is required to determine HCP understanding of support needs. Previous research including CHC patients has indicated that clinicians can underestimate the impact of blood cancers on patients' lives and psychological wellbeing (17,39). In addition, clinicians often lack the confidence, time and resources to deal with the psychosocial consequences of cancer (21,37). A need to focus on physical aspects of the disease and variation in CNS availability (40,41) could potentially result in under provision of emotional support (19).

Many sources of support discussed by patients are likely to have been impacted by the COVID-19 pandemic, due to lockdown restrictions and infection concerns, given that patients with haematological cancers are particularly vulnerable (42). This could result in some choosing to avoid face-to-face groups and services, or being encouraged to do so by HCPs. Online peer support was discussed by patients, and has been found to improve psychosocial wellbeing in cancer patients, but is recommended as complementary to face-to-face support rather than a replacement (43). Providing counselling or similar psychological support via videoconferencing has also been suggested, though barriers have been identified including: concerns regarding confidentiality, technical issues and software requirements (e.g. computer and webcam), and whether patients are able to talk openly in private, whilst in their own home (44,45).

Previous research has discussed the impact of cancer on patients' families and carers, including worsening psychological wellbeing, physical health and financial security (46–49), which may vary throughout illness (50). Suggestions to improve family outcomes include formally measuring caregiver needs, education and empowerment (51). A review of measures to assess the impact of caring found few had been systematically evaluated for cancer populations (52). There were also gaps, with few covering changing family dynamics (which we found important), and many measures being outdated, thus potentially irrelevant, especially for people living longer with cancer (52). CHCs present specific problems due to their relapsing/remitting nature and the anxiety, repeated upheaval and on-going uncertainty for patients and families. The loneliness and confusion experienced by some family members in our study suggests that peer support would be useful. A recent feasibility study found one-to-one peer support was acceptable and beneficial for caregivers of those with newly diagnosed haematological cancers (53), with similar findings for group-based peer support (54).

This study has allowed us to capture a breadth of experiences and changing attitudes by interviewing patients at varying intervals post diagnosis, and with different treatment pathways. Although prolonged time since diagnosis may affect recall, referring back to diaries and calendars is likely to have mitigated this; moreover, in many cases, patient accounts were corroborated by relatives. However, relatives were not present at every interview, a study limitation, especially with regard to views about support for carers and relatives. Our findings are likely to be transferable within the UK, and in countries with similar approaches to healthcare.

Conclusions

Individual preferences and attitudes resulted in marked differences concerning perceived needs for support, whether and how it was accessed, and the sources utilised. Relationships with others,

For peer review only - http://bmjopen.bmj.com/site/about/guidelines.xhtml

including HCPs and family members, influenced whether support was considered available by patients, and whether or not they drew on it. Preferences were also found to vary within individuals over time, mainly due to the chronic and changing nature of CHCs; the need for treatment after observation often leading to patients seeking/receiving more support. Unmet needs were also evident, especially at key points of the clinical pathway, such as diagnosis and relapse, but also more generally over time. Patients with CHCs may live with distress related to their condition for many years; thus, the monitoring of emotional needs should be considered as equally important as the monitoring of physical symptoms, particularly as the latter may in fact never occur. HCPs should be encouraged, and facilitated to discuss psychosocial issues with patients, and offer support and/or referral as appropriate. Our findings underscore the requirement for CHC patients to receive support that recognises and encompasses their psychosocial needs, and takes account of the broader context of individual patients' lives, and their use of existing networks.

Figure 1. Diagram of the impact of patients' individual approaches on identified themes

Acknowledgements

The authors are grateful to the patients who participated in interviews and shared their experiences with us.

Competing interests

None declared.

Funding

This work was supported by Cancer Research UK [29685] and the National Institute for Health Research [RP-PG-0613-2002]. No funders were involved in the study design or data collection, analysis, interpretation and reporting.

Ethics approval

Details concerning HMRN's configuration, methods and ethical approvals can be found elsewhere (55,56). For the present study, ethical approval was provided by the London, City and East Research Ethics Committee (REC:16/ LO/0740).

Patient consent for publication

Not applicable.

Data availability statement

The datasets analysed during the current study are not publicly available as participants did not provide permission for their entire interview transcripts to be published, and ethical approval was

For peer review only

For peer review only - http://bmjopen.bmj.com/site/about/guidelines.xhtml

not sought for this. A document containing the qualitative themes, and example codes and quotes is available from the authors on reasonable request.

Author contributions

DH, ER, AS and RP designed the study; AS identified potential participants. DH and DM recruited participants, DH conducted interviews. Transcripts were coded and analysed by RS with input from DH, DM and AH. RS wrote the first draft of the manuscript, which was revised by DH, DM, AH, ER, AS and RP, who also commented on clinical aspects of the study. All authors read and approved the final manuscript.

For peer review only

For peer review only - http://bmjopen.bmj.com/site/about/guidelines.xhtml

REFERENCES

1. Swerdlow S, Campo E, Harris N, Jaffe E, Pileri S, Stein H, et al. WHO Classification of Tumours of Haematopoietic and Lymphoid Tissues [Internet]. 2017 [cited 2019 Feb 8]. Available from: http://publications.iarc.fr/Book-And-Report-Series/Who-Iarc-Classification-Of-Tumours/Who-Classification-Of-Tumours-Of-Haematopoietic-And-Lymphoid-Tissues-2017

2. Sung H, Ferlay J, Siegel RL, Laversanne M, Soerjomataram I, Jemal A, et al. Global Cancer Statistics 2020: GLOBOCAN Estimates of Incidence and Mortality Worldwide for 36 Cancers in 185 Countries. CA Cancer J Clin. 2021;71(3):209–49.

3. Office for National Statistics. Cancer registration statistics, England [Internet]. 2017 [cited 2019 Oct 18]. Available from: https://www.ons.gov.uk/peoplepopulationandcommunity/healthandsocialcare/conditionsanddiseases/datasets/cancerregistrationstatisticscancerregistrationstatisticsengland

4. Haematological Malignancy Research Network. HMRN - Statistics [Internet]. 2022 [cited 2020 Apr 12]. Available from: https://www.hmrn.org/statistics

5. Howell DA, McCaughan D, Smith AG, Patmore R, Roman E. Incurable but treatable: Understanding, uncertainty and impact in chronic blood cancers—A qualitative study from the UK's Haematological Malignancy Research Network. PLOS ONE. 2022 Feb 10;17(2):e0263672.

6. Blood Cancer UK | Watch and wait [Internet]. Blood Cancer UK. 2022 [cited 2022 Jul 26]. Available from: https://bloodcancer.org.uk/understanding-blood-cancer/watch-wait/

7. Bewarder M, Stilgenbauer S, Thurner L, Kaddu-Mulindwa D. Current Treatment Options in CLL. Cancers. 2021 Jan;13(10):2468.

8. Matasar MJ, Luminari S, Barr PM, Barta SK, Danilov AV, Hill BT, et al. Follicular Lymphoma: Recent and Emerging Therapies, Treatment Strategies, and Remaining Unmet Needs. The Oncologist. 2019 Nov;24(11):e1236–50.

9. Rajkumar SV. Multiple myeloma: Every year a new standard? Hematol Oncol. 2019 Jun;37 Suppl 1:62–5.

10. Allart-Vorelli P, Porro B, Baguet F, Michel A, Cousson-Gélie F. Haematological cancer and quality of life: a systematic literature review. Blood Cancer J. 2015 Apr;5(4):e305.

11. Holtzer-Goor KM, Schaafsma MR, Joosten P, Posthuma EFM, Wittebol S, Huijgens PC, et al. Quality of life of patients with chronic lymphocytic leukaemia in the Netherlands: results of a longitudinal multicentre study. Qual Life Res Int J Qual Life Asp Treat Care Rehabil. 2015 Dec;24(12):2895–906.

12. Goswami P, Khatib Y, Salek S. Haematological malignancy: Are we measuring what is important to patients? A systematic review of quality-of-life instruments. Eur J Haematol. 2019 Apr;102(4):279–311.

For peer review only

For peer review only - http://bmjopen.bmj.com/site/about/guidelines.xhtml

13. Goswami P, Oliva EN, Ionova T, Else R, Kell J, Fielding AK, et al. Quality-of-life issues and symptoms reported by patients living with haematological malignancy: a qualitative study. Ther Adv Hematol. 2020;11:2040620720955002.

14. Waweru C, Kaur S, Sharma S, Mishra N. Health-related quality of life and economic burden of chronic lymphocytic leukemia in the era of novel targeted agents. Curr Med Res Opin. 2020 Sep;36(9):1481–95.

15. Maher K, Vries KD. An exploration of the lived experiences of individuals with relapsed Multiple Myeloma. Eur J Cancer Care (Engl). 2011;20(2):267–75.

16. Molassiotis A, Wilson B, Blair S, Howe T, Cavet J. Unmet supportive care needs, psychological well-being and quality of life in patients living with multiple myeloma and their partners. Psychooncology. 2011 Jan;20(1):88–97.

17. Evans J, Ziebland S, Pettitt AR. Incurable, invisible and inconclusive: watchful waiting for chronic lymphocytic leukaemia and implications for doctor-patient communication. Eur J Cancer Care (Engl). 2012 Jan;21(1):67–77.

18. Lamers J, Hartmann M, Goldschmidt H, Brechtel A, Hillengass J, Herzog W. Psychosocial support in patients with multiple myeloma at time of diagnosis: who wants what? Psychooncology. 2013;n/a–n/a.

19. Swash B, Hulbert-Williams N, Bramwell R. Unmet psychosocial needs in haematological cancer: a systematic review. Support Care Cancer. 2014 Apr 1;22(4):1131–41.

20. Swash B, Hulbert-Williams N, Bramwell R. ‘Haematological cancers, they’re a funny bunch’: A qualitative study of non-Hodgkin’s lymphoma patient experiences of unmet supportive care needs. J Health Psychol. 2016 Jul 28;1359105316660179.

21. Hulin C, Hansen T, Heron L, Pughe R, Streetly M, Plate A, et al. Living with the burden of relapse in multiple myeloma from the patient and physician perspective. Leuk Res. 2017 May 31;59:75–84.

22. Tsatsou I, Konstantinidis T, Kalemikerakis I, Adamakidou T, Vlachou E, Govina O. Unmet Supportive Care Needs of Patients with Hematological Malignancies: A Systematic Review. Asia-Pac J Oncol Nurs. 2020 Oct 15;8(1):5–17.

23. LeBlanc MR, LeBlanc TW, Bryant AL, Pollak KI, Bailey DE, Smith SK. A qualitative study of the experiences of living my multiple myeloma. Oncol Nurs Forum. 2021 Mar 1;48(2):151–60.

24. Rubins Z, Gibson BJ, Chantry A. What Can Patient Narratives Reveal to Us About the Experience of a Diagnosis of Myeloma? A Qualitative Scoping Review. J Patient Exp. 2022 Jan 1;9:23743735221079132.

25. El-Jawahri AR, Vandusen HB, Traeger LN, Fishbein JN, Keenan T, Gallagher ER, et al. Quality of life and mood predict posttraumatic stress disorder after hematopoietic stem cell transplantation. Cancer. 2016;122(5):806–12.

26. HMRN. HMRN - Survival [Internet]. 2022 [cited 2022 Jul 18]. Available from: https://hmrn.org/statistics/survival

For peer review only

27. Cuffe CH, Quirke MB, McCabe C. Patients' experiences of living with multiple myeloma. Br J Nurs. 2020 Jan 23;29(2):103–10.

28. Raphael D, Frey R, Gott M. Maintaining psychosocial wellbeing for post-treatment haematological cancer survivors: Strategies and potential barriers. Eur J Oncol Nurs. 2019 Feb 1;38:36–41.

29. Tong A, Sainsbury P, Craig J. Consolidated criteria for reporting qualitative research (COREQ): a 32-item checklist for interviews and focus groups. Int J Qual Health Care. 2007 Dec 1;19(6):349–57.

30. Haematological Malignancy Research Network (HMRN) [Internet]. 2007 [cited 2019 Feb 26]. Available from: https://www.hmrn.org/home

31. Patton MQ. Qualitative Research and Evaluation Methods: Integrating Theory and Practice. Fourth Edition. Thousand Oaks, CA: Sage Publications; 2015.

32. Braun V, Clarke V. One size fits all? What counts as quality practice in (reflexive) thematic analysis? Qual Res Psychol. 2021;18(3):328–52.

33. Braun V, Clarke V. Conceptual and design thinking for thematic analysis. Qual Psychol. 2022;9(1):3–26.

34. Molassiotis A, Wilson B, Blair S, Howe T, Cavet J. Living with multiple myeloma: experiences of patients and their informal caregivers. Support Care Cancer Off J Multinatl Assoc Support Care Cancer. 2011 Jan;19(1):101–11.

35. Damen MDC, Westerweel PE, Levin MD, Pelle AJ. Unmet supportive care needs, anxiety and depression in haematology patients during watch-and-wait. Psychooncology. 2022;31(2):176–84.

36. NHS England. National Cancer Patient Experience Survey [Internet]. 2021 [cited 2022 Jul 20]. Available from: https://www.ncpes.co.uk/2021-national-results/

37. Newton JC, O'Connor M, Saunders C, Moorin R, Ali S, Nowak AK, et al. The role of psychosocial support in the experiences of people living with advanced cancer: A qualitative exploration of patients' perspectives. Psychooncology. 2021;30(3):287–95.

38. Liptrott SJ, Bee P, Lovell K. Providing Telephone-Based Support for Patients With Haematological Malignancies: A Qualitative Investigation of Expert Nurses. Can J Nurs Res. 2022 Jun 1;54(2):121–33.

39. Hewison A, Roman E, Smith A, McCaughan D, Sheridan R, Patmore R, et al. Chronic myeloid leukemia: A qualitative interview study exploring disease impact from patient and practitioner perspectives [Internet]. In Review; 2022 Sep [cited 2022 Oct 3]. Available from: https://www.researchsquare.com/article/rs-2065284/v1

40. NHS National Cancer Programme. Excellence in Cancer Care: The Contribution of the Clinical Nurse Specialist [Internet]. 2010 [cited 2022 Jul 20]. Available from: https://www.macmillan.org.uk/documents/aboutus/commissioners/excellenceincancercarethecontributionoftheclinicalnursespecialist.pdf

For peer review only - http://bmjopen.bmj.com/site/about/guidelines.xhtml

41. Independent Cancer Taskforce. Achieving World-Class Cancer Outcomes - A Strategy for England 2015-2020 [Internet]. 2015 [cited 2022 Mar 23]. Available from: https://cancervanguard.nhs.uk/national-cancer-strategy/

42. Zomerdijk N, Jongenelis M, Yuen E, Turner J, Huntley K, Smith A, et al. Experiences and needs of people with haematological cancers during the COVID-19 pandemic: A qualitative study. Psychooncology. 2022;31(3):416–24.

43. Allison KR, Patterson P, Guilbert D, Noke M, Husson O. Logging On, Reaching Out, and Getting By: A Review of Self-reported Psychosocial Impacts of Online Peer Support for People Impacted by Cancer. Proc ACM Hum-Comput Interact. 2021 Apr 22;5(CSCW1):95:1–95:35.

44. Siegel A, Zuo Y, Moghaddamcharkari N, McIntyre RS, Rosenblat JD. Barriers, benefits and interventions for improving the delivery of telemental health services during the coronavirus disease 2019 pandemic: a systematic review. Curr Opin Psychiatry. 2021 Jul;34(4):434–43.

45. Connolly SL, Miller CJ, Lindsay JA, Bauer MS. A systematic review of providers' attitudes toward telemental health via videoconferencing. Clin Psychol Sci Pract. 2020;27(2):e12311.

46. Girgis A, Lambert S, Johnson C, Waller A, Currow D. Physical, Psychosocial, Relationship, and Economic Burden of Caring for People With Cancer: A Review. J Oncol Pract. 2013 Jul;9(4):197–202.

47. Pitceathly C, Maguire P. The psychological impact of cancer on patients' partners and other key relatives: a review. Eur J Cancer. 2003 Jul 1;39(11):1517–24.

48. Williams A leila. Psychosocial burden of family caregivers to adults with cancer. Recent Results Cancer Res Fortschritte Krebsforsch Progres Dans Rech Sur Cancer. 2014;197:73–85.

49. Geng H mei, Chuang D mei, Yang F, Yang Y, Liu W min, Liu L hui, et al. Prevalence and determinants of depression in caregivers of cancer patients. Medicine (Baltimore). 2018 Sep 28;97(39):e11863.

50. Kim Y, Given BA. Quality of life of family caregivers of cancer survivors. Cancer. 2008;112(S11):2556–68.

51. Berry LL, Dalwadi SM, Jacobson JO. Supporting the Supporters: What Family Caregivers Need to Care for a Loved One With Cancer. 2016;13(1):8.

52. Shilling V, Matthews L, Jenkins V, Fallowfield L. Patient-reported outcome measures for cancer caregivers: a systematic review. Qual Life Res. 2016 Aug 1;25(8):1859–76.

53. Husted Nielsen I, Piil K, Tolver A, Grønbæk K, Kjeldsen L, Jarden M. Family caregiver ambassador support for caregivers of patients with newly diagnosed hematological cancer: a feasibility study. Support Care Cancer. 2022 Aug 1;30(8):6923–35.

54. Pailler ME, Johnson TM, Zevon MA, Kuszczak S, Griffiths E, Thompson J, et al. Acceptability, Feasibility, and Efficacy of a Supportive Group Intervention for Caregivers of Newly Diagnosed Leukemia Patients. J Psychosoc Oncol. 2015 Mar 4;33(2):163–77.

55. Roman E, Kane E, Howell D, Lamb M, Bagguley T, Crouch S, et al. Cohort Profile Update: The Haematological Malignancy Research Network (HMRN)'s UK population-based cohorts. Int J Epidemiol. 2022 Feb 4;e87–94.

For peer review only - http://bmjopen.bmj.com/site/about/guidelines.xhtml

56. Smith A, Howell D, Crouch S, Painter D, Blase J, Wang HI, et al. Cohort Profile: The Haematological Malignancy Research Network (HMRN): a UK population-based patient cohort. Int J Epidemiol. 2018 Jun 1;47(3):700–700g.

For peer review only

For peer review only - http://bmjopen.bmj.com/site/about/guidelines.xhtml

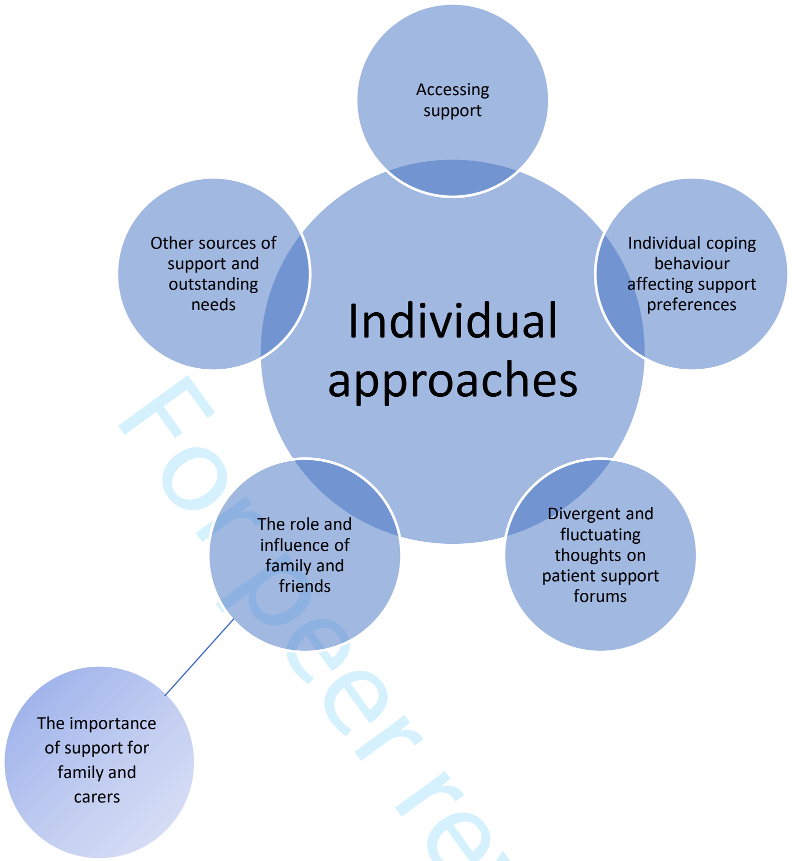

Figure 1. Diagram of the impact of patients' individual approaches on identified themes

For peer review only

Supplementary file 1

Topic guide for interviews with patients and relatives

(Tested with two patients prior to use in interviews)

Patients and relatives were asked to focus on key time-points (diagnosis, W&W, treatment), with respect to the topics included in the following three areas:

1) Understanding and impact of chronic blood cancer

- Looking back, what did you know about blood cancers, and your diagnosis specifically?
- What led you to your diagnosis initially? *(symptoms, suspected cancer?)*
- How important is it to you that you receive information about your cancer? *(why is that?)*
- How do you feel about the information given to you at diagnosis/start of treatment?
- What did you understand about your diagnosis? *(and others?)*
- What were your expectations of what would happen after diagnosis? *(treatment, follow-up)*
- What issues did you discuss at the time of your diagnosis? *(disease type, likely pathway, outcomes?)*
- What did you understand about your likely pathway and outcomes? *(immediate and later?)*
- What do you understand about your treatment?
- How do you feel about the future? *(optimistic, pessimistic?)*
- What impact has having a chronic blood cancer had on you? (activities, work, psychosocial issues)
- What strategies have you used to help you cope with your blood cancer?

2) Information

- How do you feel about getting information from HCPs generally? *(time constraints; use of language/terminology)*
- Do you feel the information given applies specifically to you? *(personalized, tailored, specific)*
- How healthcare practitioners (HCPs) ascertain your information needs?
- Is the information you received explained in a way you can understand? *(technical language; level of detail)*
- What do HCPs do to check if you understand the information they give you?
- How do you feel about asking questions? Are your questions always answered?
- Do you feel that your information needs are usually met? What worked well and could have been better? *(diagnosis; treatment initiation/cessation - examples)*
- What do you think about the timing of information from HCPs? When is the right time? *(at diagnosis; during clinic appointments; when disease status changes; at other times)*
- How do/did you feel about discussing the risks/benefits of different treatments with HCPs?
- How do you feel about discussing prognosis? *("a statement about expectations that refers to the likely course of the cancer and/or outcome") (want to know/not; timing; language)*
- What strategies do you use to absorb information? (*in general, how bad news is processed*)

3) Treatment decisions

- How do you feel about being involved in decisions with HCPs about your treatment?
- Have you been asked you if you want to be involved in decisions about treatment?
- Do you want to be involved in decisions? *(preference for patient only; clinician only; patient/clinician)*
- What should be considered during treatment decision making? *(effectiveness of treatment; side effects; prognosis; patient goals, values, preferences; impact on quality of life)*
- What might make it easier or harder for you to be involved in making decisions about your treatment? *(time; style of communication; how information is conveyed; explanations)*
- Are there particular time-points when it is harder to be involved in making decisions about treatment? *(diagnosis; treatment initiation/change; treatment cessation)*

For peer review only

Supplementary file 2
Sample details table

ID	Diagnosis[1]	Age range at interview (years)	Years since diagnosis	Known treatment line(s) preceeding interview[2,3]					
				1	2	3	4	5	6
P1	CLL	60-70	4	Observation	-	-	-	-	-
P2	MZL	60-70	15	Observation	Chemotx	Observation	-	-	-
P3	CLL	60-70	22	Observation	Chemotx	Observation	-	-	-
P4	MZL	60-70	3	Observation	Chemotx	-	-	-	-
P5	MZL	50-60	2	HPE	Observation	-	-	-	-
P6*	CLL	70-80	8	Observation	Chemotx	Observation	-	-	-
P7*	CLL	60-70	6	Observation	Chemotx	Observation	-	-	-
P8	FL	70-80	3	Chemotx	Radiotx	Observation	-	-	-
P9	CLL	80-90	5	Observation	Chemotx	-	-	-	-
P10	FL	70-80	8	Observation	Chemotx	Chemotx	Chemotx	-	-
P11	Myeloma	60-70	10	Observation	Chemotx	Observation	-	-	-
P12	MZL	70-80	5	Observation	Chemotx	-	-	-	-
P13	CLL	50-60	1	Observation	-	-	-	-	-
P14	Myeloma	60-70	4	Steroids	Radiotx	Chemotx	Chemotx	Chemotx	SCT
P15	FL	70-80	3	Observation	Chemotx	-	-	-	-
P16	Myeloma	60-70	2	Chemotx	Chemotx	Chemotx	SCT	Observation	-
P17*	FL	60-70	3	Observation	-	-	-	-	-
P18	Myeloma	60-70	3	Chemotx	Chemotx	Chemotx	SCT	Observation	
P19	FL	50-60	3	Steroids	Chemotx	Chemotx	Observation	-	-
P20*	CLL	70-80	4	Observation	-	-	-	-	-
P21*	Myeloma	70-80	3	Steroids	Chemotx	Chemotx	Chemotx	SCT	-
P22*	CLL	70-80	3	Observation	Clinical trial	Observation	-	-	-
P23	Myeloma	60-70	3	Observation	-	-	-	-	-
P24	FL	50-60	4	Steroids	Chemotx	Radiotx	Observation	-	-
P25	FL	60-70	4	Chemotx	Chemotx	-	-	-	-
P26	Myeloma	70-80	4	Observation	-	-	-	-	-
P27*	CLL	70-80	4	Chemotx	Observation	-	-	-	-
P28	Myeloma	60-70	4	Steroids	Chemotx	Chemotx	SCT	Clinical trial	Chemotx
P29	CLL	70-80	3	Clinical trial	Observation	-	-	-	-
P30*	Myeloma	70-80	2	Observation	-	-	-	-	-
P31*	Myeloma	70-80	2	Radiotx	Steroids	Chemotx	Observation	-	-
P32*	MZL	60-70	2	Observation	Chemotx	Observation	-	-	-
P33	Myeloma	50-60	3	Chemotx	Chemotx	SCH	Observation	-	-
P34	FL	50-60	4	Steroids	Chemotx	Chemotx	Chemotx	-	-
P35	Myeloma	50-60	2	Chemotx	Chemotx	Chemotx	Chemotx	SCT	Observation

[1] CLL – Chronic Lymphocytic Leukaemia; FL – Follicular Lymphoma; MZL – Systemic Marginal Zone Lymphoma.
[2] Chemotx = Chemotherapy; HPE = H. Pylori eradication; Radiotx = Radiotherapy; SCT = Stem cell transplant (all autografts); SCH = Stem cell harvest (shown as SCT did not take place).
[3] Does not include supportive care (e.g. blood product transfusions, plasma exchange, bisphosphonates, cell mobilization products)
*Relative present at interview

For peer review only

COREQ (COnsolidated criteria for REporting Qualitative research) Checklist

No. Item	Guide questions/description	Page	Further detail
**Domain 1: Research team and reflexivity**			
**Personal characteristics**			
1. Inter viewer/facilitator	Which author/s conducted the interview or focus group?	5	DM
2. Credentials	What were the researcher's credentials? E.g. PhD, MD	n/a	BA, RN, MSc
3. Occupation	What was their occupation at the time of the study?	n/a	Senior Research Fellow
4. Gender	Was the researcher male or female?	5	Female
5. Experience and training	What experience or training did the researcher have?	n/a	Registered Nurse, conducted many qualitative interviews.
**Relationship with participants**			
6. Relationship established	Was a relationship established prior to study commencement?	n/a	No
7. Participant knowledge of the interviewer	What did the participants know about the researcher? e.g. personal goals, reasons for doing the research	n/a	No knowledge.
8. Interviewer characteristics	What characteristics were reported about the interviewer/facilitator? e.g. Bias, assumptions, reasons and interests in the research topic	n/a	Experienced qualitative health services researcher.
**Domain 2: Study design**			
**Theoretical framework**			
9. Methodological orientation and Theory	What methodological orientation was stated to underpin the study? e.g. grounded theory, discourse analysis, ethnography, phenomenology, content analysis	5	Reflexive thematic analysis.
**Participant selection**			
10. Sampling	How were participants selected? e.g. purposive, convenience, consecutive, snowball	5	Purposive.
11. Method of approach	How were participants approached? e.g. face-to-face, telephone, mail, email	5	Mail, followed up by telephone if expressed interest.
12. Sample size	How many participants were in the study?	5	35
13. Non-participation	How many people refused to participate or dropped out? Reasons?	n/a	None dropped out. 20 patients did not respond to invite for reasons unknown.
**Setting**			
14. Setting of data collection	Where was the data collected? e.g. home, clinic, workplace	5	Home, University.
15. Presence of non-participants	Was anyone else present besides the participants and researchers?	5	Relatives were present at 10 interviews.
16. Description of sample	What are the important characteristics of the sample? e.g. demographic data, date	5	Also found in Supplementary file 2.
**Data collection**			

For peer review only

17. Interview guide	Were questions, prompts, guides provided by the authors? Was it pilot tested?	5	Topic guide found in Supplementary file 1.
18. Repeat interviews	Were repeat inter views carried out? If yes, how many?	n/a	No repeat interviews were carried out.
19. Audio/visual recording	Did the research use audio or visual recording to collect the data?	5	Audio.
20. Field notes	Were field notes made during and/or after the interview or focus group?	n/a	No.
21. Duration	What was the duration of the inter views or focus group?	5	60-90 minutes
22. Data saturation	Was data saturation discussed?	n/a	Yes, interviews were continued until saturation.
23. Transcripts returned	Were transcripts returned to participants for comment and/or correction?	n/a	No transcripts were returned.
**Data analysis**			
24. Number of data coders	How many data coders coded the data?	5	RS, DM, DH, AH
25. Description of the coding tree	Did authors provide a description of the coding tree?	Figure 1	
26. Derivation of themes	Were themes identified in advance or derived from the data	F5	Data manged in Microsoft Word.
27. Software	What software, if applicable, was used to manage the data	n/a	No software was used.
28. Participant checking	Did participants provide feedback on findings?	n/a	No.
**Reporting**			
29. Quotations presented	Were participant quotations presented to illustrate the themes/findings? Was each quotation identified? e.g. participant number	6-11	Quotations identified by participant number.
30. Data and findings consistent	Was there consistency between the data presented and the findings?	6-11	Yes.
31. Clarity of major themes	Were major themes clearly presented in the findings?	6-11, Figure 1	Major themes were presented alongside subthemes.
32. Clarity of minor themes	Is there a description of diverse cases or discussion of minor themes?	6-11	Yes, a range of responses are considered.

Developed from: Tong A, Sainsbury P, Craig J. Consolidated criteria for reporting qualitative research (COREQ): a 32-item checklist for interviews and focus groups. *International Journal for Quality in Health Care*. 2007. Volume 19, Number 6: pp. 349 – 357

For peer review only

Experiences and preferences for psychosocial support: A qualitative study exploring the views of patients with chronic haematological cancers

Journal:	*BMJ Open*
Manuscript ID	bmjopen-2022-070467.R1
Article Type:	Original research
Date Submitted by the Author:	05-Jun-2023
Complete List of Authors:	Sheridan, Rebecca; University of York, Health Sciences<br>McCaughan, Dorothy; University of York, Health Sciences<br>Hewison, Ann; University of York, Health Sciences<br>Roman, Eve; University of York, Health Sciences<br>Smith, Alexandra; University of York, Health Sciences<br>Patmore, Russell; Castle Hill Hospital<br>Howell, Debra; University of York, Health Sciences
**Primary Subject Heading**:	Haematology (incl blood transfusion)
Secondary Subject Heading:	Health services research, Qualitative research
Keywords:	Leukaemia < HAEMATOLOGY, Lymphoma < HAEMATOLOGY, Myeloma < HAEMATOLOGY

For peer review only - http://bmjopen.bmj.com/site/about/guidelines.xhtml

I, the Submitting Author has the right to grant and does grant on behalf of all authors of the Work (as defined in the below author licence), an exclusive licence and/or a non-exclusive licence for contributions from authors who are: i) UK Crown employees; ii) where BMJ has agreed a CC-BY licence shall apply, and/or iii) in accordance with the terms applicable for US Federal Government officers or employees acting as part of their official duties; on a worldwide, perpetual, irrevocable, royalty-free basis to BMJ Publishing Group Ltd ("BMJ") its licensees and where the relevant Journal is co-owned by BMJ to the co-owners of the Journal, to publish the Work in this journal and any other BMJ products and to exploit all rights, as set out in our licence.

The Submitting Author accepts and understands that any supply made under these terms is made by BMJ to the Submitting Author unless you are acting as an employee on behalf of your employer or a postgraduate student of an affiliated institution which is paying any applicable article publishing charge ("APC") for Open Access articles. Where the Submitting Author wishes to make the Work available on an Open Access basis (and intends to pay the relevant APC), the terms of reuse of such Open Access shall be governed by a Creative Commons licence – details of these licences and which Creative Commons licence will apply to this Work are set out in our licence referred to above.

Other than as permitted in any relevant BMJ Author's Self Archiving Policies, I confirm this Work has not been accepted for publication elsewhere, is not being considered for publication elsewhere and does not duplicate material already published. I confirm all authors consent to publication of this Work and authorise the granting of this licence.

For peer review only

Experiences and preferences for psychosocial support: A qualitative study exploring the views of patients with chronic haematological cancers

AUTHORS: Rebecca Sheridan, Dorothy McCaughan, Ann Hewison, Eve Roman, Alexandra Smith, Russell Patmore, Debra Howell

CORRESPONDING AUTHOR:

Debra Howell
Epidemiology & Cancer Statistics Group, Department of Health Sciences, Seebohm Rowntree Building, University of York, York, UK. YO10 5DD.
Telephone: +44 (0) 904 321895
Email: debra.howell@york.ac.uk

CO-AUTHORS

Rebecca Sheridan
Epidemiology & Cancer Statistics Group, Department of Health Sciences, Seebohm Rowntree Building, University of York, York, UK. YO10 5DD.
Telephone: +44 (0) 904 321079
Email: rebecca.sheridan@york.ac.uk

Dorothy McCaughan
Epidemiology & Cancer Statistics Group, Department of Health Sciences, Seebohm Rowntree Building, University of York, York, UK. YO10 5DD.
Telephone: +44 (0) 904 321348
Email: dorothy.mccaughan@york.ac.uk

Ann Hewison
Epidemiology & Cancer Statistics Group, Department of Health Sciences, Seebohm Rowntree Building, University of York, York, UK. YO10 5DD.
Telephone: +44 (0) 904 321916
Email: ann.hewison@york.ac.uk

Eve Roman
Epidemiology & Cancer Statistics Group, Department of Health Sciences, Seebohm Rowntree Building, University of York, York, UK. YO10 5DD.
Telephone: +44 (0) 904 321882
Email: eve.roman@york.ac.uk

Alexandra Smith
Epidemiology & Cancer Statistics Group, Department of Health Sciences, Seebohm Rowntree Building, University of York, York, UK. YO10 5DD.
Telephone: +44 (0) 904 321884

For peer review only

For peer review only - http://bmjopen.bmj.com/site/about/guidelines.xhtml

Email: alexandra.smith@york.ac.uk

Russell Patmore
Queens Centre for Oncology and Haematology, Castle Hill Hospital, Cottingham, UK.
Telephone: +44 (0) 482 604444
Email: russell.patmore@nhs.net

KEYWORDS: Haematological cancer; psychosocial; peer support; multiple myeloma; leukaemia; lymphoma

WORD COUNT: 5662, including all quotations

For peer review only

For peer review only - http://bmjopen.bmj.com/site/about/guidelines.xhtml

ABSTRACT

Objectives Patients with chronic haematological cancers are often treated on a relapsing-remitting pathway, which may extend for many years. Such diagnoses are associated with uncertainties that often cause anxiety and distress, meaning patients (and families) are susceptible to potentially prolonged emotional difficulties, across the cancer journey. Experiences and preferences regarding psychosocial needs and support over time are relatively unexplored, which this study aimed to address.

Setting and design Set within the UK's Haematological Malignancy Research Network (HMRN: an ongoing population-based cohort that generates evidence to underpin improved clinical practice) a qualitative, exploratory study was conducted, utilising semi-structured interviews. Reflexive thematic analysis was used to assess the interview data via an exploratory, inductive approach, underpinned by the research questions.

Participants Thirty-five patients were included with chronic lymphocytic leukaemia, follicular lymphoma, marginal zone lymphoma, or myeloma; 10 of whom were interviewed alongside a relative.

Results Five themes were identified from the data: (i) accessing support; (ii) individual coping behaviour affecting support preferences; (iii) divergent and fluctuating thoughts on patient support forums; (iv) the role, influence and needs of family and friends; and (v) other sources of support and outstanding needs. Findings suggest that patients' individual attitudes towards support varied over time. This also influenced whether support was perceived to be available, and if it was then utilised.

Conclusion This study highlighted the variation in preferences towards psychosocial support among patients with chronic haematological cancers. As patients can live for many years with significant emotional difficulties, they may benefit from frequent monitoring of their psychosocial wellbeing, as well as signposting to holistic support, if this is needed.

ARTICLE SUMMARY

Strengths and limitations of this study

- Comprehensive findings due to inclusion of patients with a range of chronic haematological cancers, demographic characteristics and treatment pathways.
- Robust data collection and analysis conducted by an experienced team of qualitative researchers.
- Transferable findings within the UK and countries with similar healthcare systems.
- Potential recall difficulties due to time since diagnosis, despite the use of diaries/calendars.
- Limited inclusion of relatives/carers (due to patient preferences) and patients under 50 years of age (due to disease rarity); hence the need for further research with these groups.

For peer review only - http://bmjopen.bmj.com/site/about/guidelines.xhtml

For peer review only

INTRODUCTION

Haematological malignancies (blood cancers) are a diverse group with many sub-types (1). Broadly classified as leukaemias, lymphomas and myeloma, these are collectively the fifth most common cancer globally (2), with increased incidence amongst ageing populations (3,4). While some of these malignancies are potentially curable, the majority (around 60%) are not, in which case treatments aim to ameliorate symptoms and slow progression (5). The four most common chronic haematological cancers (CHCs) are chronic lymphocytic leukaemia (CLL), follicular lymphoma (FL), marginal zone lymphoma (MZL) and myeloma. Some patients with such conditions may be treated from diagnosis; while others will be managed on 'watch and wait', either never requiring treatment, or alternating between treatment and observation with the aim of restoring remission (6). Treatments may include combinations of chemotherapy, radiotherapy, stem cell transplant and targeted agents (7–9).

Patients with CHCs may be successfully managed on relapsing-remitting pathways over many years, though symptom burden and loss of functionality can cause increased dependence on others, and may have a detrimental effect on quality of life (10–12). Living with a CHC may be associated with increased emotional and psychological distress, linked primarily to uncertainty concerning disease progression/relapse, treatment, and prognosis, compounded by poor diagnostic understanding, and informational deficiencies (5,13–22). Accordingly, the most commonly reported unmet supportive care needs discussed by CHC patients are informational and psychological (23). The importance of timely psychosocial support during observation, and before, during, and after treatment is underscored in a prospective, longitudinal study, which notes an increased risk of patients developing psychological disorders when supportive needs are not met (24).

CHC patients' experiences of accessing and receiving support remain relatively unexplored, despite the growing significance of this issue due to improved outcomes requiring long-term follow-up; five-year relative survival estimates range from 48% (myeloma), to 88% (FL) (25). Relevant literature emphasises the value patients place on social support, which also extends to family and significant others, to enable adjustment and coping (13,18,26,27). Patients may also rely on peer support groups and health care professionals (HCPs), such as clinical nurse specialists (CNSs), for emotional as well as informational support (18,27).

Understanding patients' views of seeking and receiving support, and associated barriers and facilitators, is circumscribed by the small-scale of many published studies, predominantly focusing on patients with myeloma (13,14,19,21,26). Our own study therefore elicited in-depth perspectives from a comparatively large qualitative sample of thirty-five patients with CLL, FL, myeloma and MZL. Data were explored with the aim of examining the experiences of seeking and receiving emotional and psychological support, from the point of diagnosis onwards, and identifying perceived needs and preferences.

METHODS

This study is part of a broader programme of work examining the perspectives of CHC patients concerning information needs and treatment decisions. Further details can be found elsewhere (5). Methods and results are reported according to the Consolidated Criteria for Reporting Qualitative Research guidelines (28).

For peer review only - http://bmjopen.bmj.com/site/about/guidelines.xhtml

Study design and setting

A qualitative, exploratory study was conducted, using semi-structured interviews. It was set within the UK's Haematological Malignancy Research Network (HMRN: https://hmrn.org) (29); a population-based cohort that was established in 2004 across an area of 4 million people with similar socio-demographic characteristics to the UK as a whole (30). HMRN is an on-going collaboration between university academics, National Health Service (NHS) clinicians, and patients and carers, who collectively co-design and conduct research, with the aim of generating evidence that can be used to improve clinical practice, locally, nationally and internationally.

Sampling

Sampling was purposive; we aimed to capture a range of experiences relating to the topic of interest (31). Participants with CLL, FL, myeloma or MZL were selected according to the median diagnostic age for each cancer, with variation by gender, time since diagnosis, and events on the clinical pathway; an overview of patient characteristics can be found in Supplementary file 1. Refinement of the sampling strategy led to inclusion of patients across broader demographic categories; for example, by including those above and below sub-type median diagnostic age.

Data collection

Checks with patients' clinical teams ensured they were well enough to participate in the study. Identified individuals were sent information and the researcher's contact details to discuss participation. Interviews were conducted by an experienced female researcher (DM), between February-October 2019, at a time and place of the patient's choosing, usually their home, with a friend or relative present if desired. Interviews continued until data saturation had been reached, defined as the point at which no new insights could be added to the analysis (32). Participants were invited to ask questions about the study prior to interview, and assurances were given regarding anonymity and confidentiality. Written consent was obtained from all participants, including for the use of direct quotations. Interviews lasted 60-90 minutes, and were digitally audio-recorded. A semi-structured topic guide (Supplementary file 2), developed from the extant research literature (15–17,33,34), and in conjunction with haematology practitioners, was used flexibly to guide discussions, which were subsequently transcribed verbatim, and checked, corrected and pseudonymised by the interviewer.

Data analysis

Reflexive thematic analysis was used, an approach more akin to a transtheoretical technique, than a methodology predicated on a particular theoretical stance (35,36). Analysis was carried out by an experienced team, using an exploratory, inductive approach, underpinned by the research questions. Initial codes were developed by the first author (RS), with further coding and development of themes being a collaborative process involving RS, DM, DH and AH. This was based on active searching for patterns of meaning across the data set, achieved through the dual processes of immersion in the data, and distancing, to allow time for reflection and the development of insights. Regular meetings (RS, DM, DH, AH) focussing on theme review and refinement, supplemented by written memos and reflections, promoted critical thinking, and enhanced reflexivity and interpretive depth. The overall aim was to 'interrogate' the data to arrive at an interpretation that was *'insightful, thoughtful, rich…nuanced'* (36). Patient quotes are presented in italics and refer to participant numbers (e.g. P1 for the patient and P1R for the patient's relative).

For peer review only - http://bmjopen.bmj.com/site/about/guidelines.xhtml

Patient and public involvement

Patients and relatives who regularly attended a blood cancer support group were involved in prioritising the study aims, preparing the funding application and disseminating findings. Long-standing links between the research team and members of this support group facilitate their active involvement in HMRN's activities, although these patients and relatives did not take part in the interviews.

RESULTS

Thirty-five patients participated in the study; ten relatives contributed to interviews. Twelve patients had myeloma, 10 CLL, 8 FL and 5 MZL; treatment pathways varied (Supplementary file 1). Twenty-two patients were aged ≥60 years; and 19 were male.

Five themes were identified: (i) accessing support; (ii) individual coping behaviour affecting support preferences; (iii) divergent and fluctuating thoughts on patient support forums; (iv) the role, influence and needs of family and friends; (v) other sources of support and outstanding needs. Individual preferences were evident in each theme, with the patient's personality impacting their use of support (Figure 1).

Theme 1: Accessing support

Support was available from a number of sources, but was impacted by the patient's diagnosis and awareness of the resources available, informed by their own research and/or HCP knowledge and advice.

Subtheme 1.1 Varied types of support

Patients reported accessing or awareness of various types of peer support, including face-to-face/online forums, and being introduced to, or knowing, other patients who shared their diagnosis. The majority of face-to-face support groups were not disease specific, but accommodated people with any haematological cancer. Online cancer specific groups referenced included Myeloma UK, CLL Support Association and also Facebook groups.

Informal support from family and friends was particularly important, whilst nurses, and, sometimes doctors, also contributed. Patients utilised NHS and private counselling services and complementary therapies such as acupuncture. Macmillan centres were attended by some, as well as support days and events hosted by charities, such as Myeloma UK.

Subtheme 1.2 Knowing where to find support

Most patients knew about online and face-to-face support groups, even if they did not want to use these resources. It was not always clear how patients became aware of support groups; some were told by their doctor or nurse (P10, P12, P28), or received information via leaflets (P5). P19 and P3 specifically noted finding websites/forums based in America; P19 found *'it wasn't anything particularly helpful'* due to their non-UK focus.

Some patients contacted others who shared their diagnosis via their GP or family and friends. For example, P14's GP organised for someone with the same condition to speak to him: *'hearing from somebody who's been through it all and got the badge certainly helped'.* P33 had requested a

For peer review only - http://bmjopen.bmj.com/site/about/guidelines.xhtml

'buddy', something P21R would have welcomed for herself and her relative: *'someone that's like a year in to having this, who would sit down and explain what they discovered and how it works, and what to expect'.*

Some patients (P1, P4) requested counselling, while others (P9, P13) were offered a referral by nursing staff. Nurses also informed some patients (P8, P25) about other measures that might help, such as therapeutic massage. P16 had not been told about Macmillan support centres, but would have found this useful; it was unclear whether other patients were informed about them.

Subtheme 1.3 Impact of disease subtype on accessing peer support

A number of patients commented on difficulties due to their cancer subtype, such as its rarity (P5, P25, P33). Because of this, P25 felt *'on me [sic] own'* and unsupported, noting that neither her nor her daughter could find any support groups. This could be problematic even for patients who shared the same diagnosis; P5 explained people at her support group all had lymphoma or leukaemia but she didn't perceive herself as having the same disease due to differences in site, clarifying: *'I have [the same cancer] but it's in my stomach'.*

Theme 2: Individual coping behaviour affecting support preferences

The patient's personal approach to their cancer influenced their coping strategies, often resulting in a reluctance to access formal support.

Subtheme 2.1 Perceptions of illness: Distancing, distraction, fighting back and maintaining hope

A number of patients didn't want to *'dwell'* on their cancer and discussed measures taken to actively distance or distract themselves. Instead of seeking specific support, such individuals focused on other activities, with P17 saying: *'We have quite a lot of interests, so we're always doing things and I think that's good because it just takes your mind off things'.* P27 and P29 took a philosophical approach to their cancer: *'there's nothing you can do about it…you make the best…of what is available for you'* (P27). Patients did not want to be defined by their condition, or for their condition to *'rule [their] life'* (P3). P5 attended a support group infrequently, as they didn't want their cancer to *'represent'* them. Similarly, P35 commented: *'for some people [at support group] it's like their whole life is having myeloma. I just don't want to be like that'.*

Many patients highlighted specific coping strategies to distract themselves from their CHC, including exercising, dog walking and gardening. Having another focus was important to P3 when trying to accept their diagnosis and deferred treatment (watch and wait): *'[running] made a massive difference because I'm doing something…I'm fighting back'.*

Some people felt they needed to stay positive and maintain hope with respect to their cancer and future outcomes. P24 adopted a *'positive mental attitude';* P25 described focussing on future events as *'summit to aim for'.* P13 felt that support group attendance might interfere with their positive approach due to the sharing of negative experiences, explaining: *'I wouldn't want to hear all of the things, you know, "well, it did this to me and it did that to me, and I thought I was on death's door", …I need to keep buoyant and positive about it'.*

For peer review only - http://bmjopen.bmj.com/site/about/guidelines.xhtml

Subtheme 2.2 Perceptions of personality

Some patients felt they were not the 'type' to attend a support group: *'I am just a bit introverted meself [sic]…I'm not a kind of a support group type person…'* (P21). Similarly, P27 described such meetings as *'not for me'*, but did acknowledge that this could change in the future. Whilst P10 appreciated that support groups could be useful for others, they found them unnecessary due to them being inherently positive: *'I have a positive attitude and I don't feel I need support'.*

Theme 3: Divergent and fluctuating thoughts on patient support forums

Patients referred to both the significant benefits and also drawbacks of face-to-face and online support forums, with variations noted between individuals.

Subtheme 3.1 Varied perspectives

From a positive perspective, support groups were described as *'very valuable'* (P1); P6 described their group leader as their '*rock'.* Meeting others in a similar situation provided patients with reassurance and comradery: *'you're not on your own but to know somebody else is going through it, and you can tell her your worries'* (P3). P33 also noted that their efforts to maintain positivity within support groups had helped others, saying that members had remarked: *'you've cheered me up and made me feel positive because you're always so positive'.*

Whilst positive experiences were welcomed, hearing negative accounts could be frightening, reflecting patients' desires not to be exposed to these stories (see subtheme 2.1). For example, P35 explained: '*I thought I'd find [support group] terrifying, which I sort of do really because it's like people just talking about their terrible stories a lot of the time'.* P7 highlighted that patients may hear both positive and negative accounts, with the negative making them feel like they were '*in a queue sat there waiting to die'.*

Subtheme 3.2 Changing preferences over time

Patients commented on their decisions regarding when to seek group support, often depending on the status of their cancer. For example, some patients (P4, P8) did not attend face-to-face meetings during treatment due to fatigue, illness or infection risk. Engagement could increase when they needed information or advice, such as when making treatment decisions. As explained by P3, who became more active on internet forums at relapse: *'A lot of people were saying, yeah I was the same as you…I just wanted to forget about it but then when you have something to remind you that you've still got the condition a lot of people start to be more active'.* Some patients on observation didn't feel a support group was necessary as they were '*coping well'* (P17), or because their cancer didn't '*affect [their] lifestyle'* (P20).

Subtheme 3.3 Information provision

Patients generally found support groups and websites a useful source of information, and some noted that they themselves had provided information to others (P6, P13, P19). On websites in particular, patients could choose what information they wanted to engage with, and when. For example, P19 found negative material unwelcome saying: *'it used to scare me'.* P3 highlighted the importance of understanding that *'everyone is different'*, explaining: *'you have to decide what suits*

For peer review only - http://bmjopen.bmj.com/site/about/guidelines.xhtml

you, what you feel comfortable with and learn to take the advice that's useful for you and ignore the advice that's less useful'.

Theme 4: The role, influence and needs of family and friends

All patients discussed their family and friends, largely referring positively to the support these people provided. The impact of the patients' CHC on family and friends was also considered, the importance of which was recognised by patients.

Subtheme 4.1 Emotional and practical support: Reassurance, companionship and the importance of family and friends

Family and friends provided significant emotional support to the majority of patients; for example, P2 commented: *'the people who are close to you are very important because they can keep you going…I think they're very important in reassuring you'*. Patients also acknowledged the importance of practical support, including relatives or friends accompanying them to hospital appointments, reminding them what was discussed, and learning about their condition. P9 explained '*my daughter knows as much about my treatment as I do. She keeps really up-to-date with it and she's with me most treatments'.* Contrastingly, P3 attended appointments alone, because his wife would *'worry herself silly',* as appointments were *'more stress inducing'* for her than for himself. P34, who attended appointments alone due to his wife's work commitments, acknowledged that, with hindsight, practical support would have been useful.

Emotional and practical input from friends was particularly important for patients without immediate family support. P8, who lived alone, described her friend as '*marvellous',* explaining: *'I haven't got any family…She was just there for me the whole time'.* P33, whose partner had died, felt the need for practical and emotional support: *'I have to do all my own research, all my own ringing around and sometimes I think I just wish I had that person…I want somebody just to hold my hand and go, I'm going to sort that for you'.* For some, having family did not guarantee support, as exemplified by P25, who relied on a friend to provide emotional and practical help, despite living with relatives.

Subtheme 4.2 Disease characteristics and family dynamics: Impact of the cancer, perceived response of others and life context

Patients' decisions about disclosing their CHC impacted available support, and were often influenced by factors relating to their cancer. When patients felt their diagnosis would impact their lives, they often chose to tell family and friends. For example, P22 explained: *'we knew we were always going to always be at hospital…so we took the road that we would tell them'.* In contrast, when patients didn't feel their cancer was noticeably impacting them, for example when on observation, they did not always disclose their diagnosis, thus eliminating opportunities for support. P2 said: *'close family knew about it and close friends and that was it because I could get on with me [sic] life. I could manage it. Nobody needed to know'.* P20 also highlighted the nature of 'watch and wait', explaining they did not inform people about their diagnosis as: *'they wouldn't understand that there's no treatment required'.*

For peer review only - http://bmjopen.bmj.com/site/about/guidelines.xhtml

Patients hesitated to disclose their CHC to children for various reasons including their young age, because they were undertaking important exams, and/or because they did not wish to be '*a burden*' (P32). P35 knew she had to tell her children as her diagnosis was *'a big life changing event'* but was unsure about the timing, explaining: '*I just wanted to know if I could carry on my life without telling them'.* Being on watch and wait allowed P11 to keep their diagnosis from their children, who they believed were too young to understand/cope.

Even where family and friends were informed about the diagnosis, feelings were not always shared. For example, P7R explained that they did not discuss their worries in order to '*protect'* family. Similarly, P5 felt they had to be strong for others, so were providing, rather than receiving, support. Some patients did not share their feelings about their CHC even with close family, or were reluctant to ask for help, so could not be supported. For example, P2 described shock at their diagnosis, but his wife had said: *'the trouble was you didn't tell me all this so I couldn't be involved'.* P25 described feeling lonely, but wanted to *'make it easier for them [family]'*, admitting: *'I won't ask nobody for help. If somebody, if me [sic] cousin had offered to come, I would have said, "no I'm fine"'.*

Family dynamics could change as a result of the CHC diagnosis. P19 said: *'I didn't feel well and for the first time ever, my family just took full control of everything and normally I'm the one that's like the matriarch'.* However, whilst the patient was grateful, the diagnosis and changing circumstances were not accepted by everyone: *'[Daughter] wouldn't even talk about it and to this day she doesn't really. She just, you know, she just likes her mum to be mum, and functional'.*

Subtheme 4.3: The importance of support for family and carers

This was discussed by several patients, with P12 explaining they felt support was '*just as important for the family as it is for the patient'.* Patients appreciated that family are also impacted by their diagnosis; P11 suggested '*it's probably harder on my wife or my children'.* Similarly, P28 remarked that: '*The worry is done by the carers'.* In contrast, P6 acknowledged differences in perceptions of burden between the patient and their family: *'Mentally, [husband] forgets sometimes and he admits – "I forget you've got leukaemia". But it lives with me 24/7. I never forget that I've got it'.*

Patients' families found support in various settings. For example, P12 and P13 described nurses offering support to relatives, with P12 commenting that speaking to the nurse '*kind of settled [wife] a bit'.* P11 encountered another patient, also with myeloma, and explained how their wives then talked and found this useful: '*[Wife (of P11)] was a bit left in the dark she felt, so she really enjoyed talking to [contact's wife] about how she felt'.* Support was also available via dedicated Cancer Centres, with P18's partner accessing counselling and a therapeutic course. P28 praised Myeloma UK for their approach to supporting both patients and carers, including holding sessions specifically for carers at their events.

In contrast, some family members commented that their support needs were unmet. P7R described there being no information about support for relatives, saying '*it's lonely',* and highlighting that the carer also has a *'burden'.* P21R felt unsupported, suggesting counselling should be provided for patients and relatives. Sometimes support was available, but relatives were unwilling to accept it. For example, P19 commented that her daughter really needed support but wouldn't access it: *'the Macmillan nurse was really good and she offered to speak to [daughter], and she tried to ring her and she didn't want to talk about it'.*

For peer review only - http://bmjopen.bmj.com/site/about/guidelines.xhtml

Theme 5: Other sources of support and outstanding needs

Support from HCPs and other formal sources, such as counselling services, were discussed by some, with wide variation in the perceived usefulness of these resources. Unmet emotional needs were evident, however, particularly at significant time points in the patient's journey.

Subtheme 5.1 Perceptions of support from healthcare professionals

Nursing staff were considered a valuable source of support, often providing reassurance and encouragement for patients. For example, P14 said: '*[Nurse] has been there for me and guided me, cajoled me, pointed me in the right direction'.* There were differences in the type of support patients perceived nurses as providing; P5 described it as *'medical'* but '*not necessarily psychological'*, whilst P7 said: *'As well as your physical condition [nurses] were bothered about my emotional condition…'.*

A number of patients (P1, P16, P34) reported support from nurses as reliable and always available. In contrast, others were reluctant to access such input; P4 explained: *'I didn't feel I could be ringing [nurse] every day…because obviously she's got lots of other things to do and lots of other people to deal with'*. P4 acknowledged most nurses were *'understanding'*, but felt some trivialised her concerns: '*you'd ring up and they'd sort of brush it off'.* P4 and P33 expressed disappointment when nurses reneged on their stated intentions, including referral for counselling, and providing reassurance to family members.

Interactions with nursing staff appeared to vary according to CHC management, with patients on treatment (attending regularly) having greater contact than those being observed on watch and wait (attending less frequently). For example, a number of patients with myeloma (P11, P14, P18, P28), in frequent contact with nurses during treatment, discussed the importance of receiving regular support: '*we've had a lot of dealing with [nurses] and they're spot on'* (P11). Similarly, P3 said: *'The only time I can remember talking to nurses a lot was when I had my treatment and of course they were absolutely superb'.* Preferences were found to vary among those given contact details for nursing staff during periods of observation, with P2 choosing not to use these; while others appreciated this information and felt support was available if needed (P13, P17, P26). Contrastingly, P13 (solely observed since diagnosis) perceived nurses as providing '*holistics…mind, body and soul'* care to those on watch and wait, in order to *'free up the doctors, so they can see the patients that maybe need a little bit of extra time'*.

Whilst most comments about HCP support were about nurses, some patients referred to support from doctors, which was mainly, but not uniformly, perceived positively. P9 felt their doctor was *'very supportive and helpful'* when they were upset about needing chemotherapy, and P12 felt their doctor had '*plenty of empathy'.* In contrast, P4 described their doctors as speaking on a *'consultant level, with no emotions'.* A number of patients highlighted positive interactions with their GPs, with P30 describing theirs as *'fantastic'.* However, others (P6, P13, P23, P25) felt their GP lacked knowledge about their CHC. Three patients mentioned receiving support from other HCPs outwith the NHS, including nurses from Myeloma UK (P29) and Macmillan (P13, P19).

Subtheme 5.2 Counselling and complementary support

Seven patients discussed their experiences of counselling, with perceptions varying markedly. P1 felt this had been useful, facilitating the realisation that they had '*experienced a tremendous trauma'*, which had impacted their behaviour in response to treatment and infection fears. Conversely, P25 felt counselling was '*a complete waste of time'* and not what she had expected, explaining: *'To me, a*

For peer review only - http://bmjopen.bmj.com/site/about/guidelines.xhtml

counsellor is somebody that will counsel you and advise you. She never opened her mouth'. Nevertheless, P25 did feel the service had helped her '*come to terms*' with her diagnosis. P33 felt their psychologist only cared how they felt about their treatment, and not their wellbeing more generally. P35 described both positive and negative aspects of counselling, explaining that whilst it was '*helpful'* it could also be upsetting to discuss their feelings.

Four patients (P9, P16, P19, P22) referred to Macmillan services, all positively, though P22 felt these weren't aimed at her as she wasn't *'terminal'*. A number of patients discussed complementary medicines including acupuncture (P9), therapeutic massage (P8, P25) and reflexology (P23). P2 talked at length about the reassurance, support and holistic care provided by the homeopath he had attended for several years, comparing interactions to those with their doctor: *'I used to get different doctors and you just felt as though you were part of a process but…my homeopath I go to, we sit and we talk about my symptoms, how I'm feeling'.*

Subtheme 5.3 Unmet emotional needs

Some patients highlighted unmet emotional support needs. P4, who was waiting for counselling, noted: '*I didn't feel like there was anybody professional shall I say, who I could talk to and just tell them how I felt and that I was really scared...'*. P25 commented that: *'[leaflets] could do [to include] a little bit more on your thoughts. Yeah on your mind, on the emotional side of it',* whilst P5 felt they would cope with their CHC psychologically, but '*at a price'.*

A number of patients identified key points in the CHC trajectory which caused emotional turmoil, but where they felt their needs were not met. Many highlighted the *'shock'* of diagnosis, which was described as *'a huge emotional rollercoaster'* (P7), while P13 talked about becoming upset when she discovered '*lumps'* which could indicate disease progression; others described feeling shock at being told they would need treatment after a period of observation (P3, P15). Despite the significance of these issues, patients identified a lack of emotional support at these critical junctures in their lives. Concerns could also be continually present, for example patients alluded to the difficulty of living with the uncertainty caused by their cancer diagnosis: '*it will flare up again...that causes anxiety… you don't know when…it's lurking there'* (P4).

DISCUSSION

This study provides novel in-depth insights about the experiences of CHC patients in seeking, accessing and engaging with psychosocial support. Many patients relied on support groups, which were generally perceived positively, but there were also significant barriers to their use, including: distress on hearing negative stories, lack of relevant groups due to the rarity of the cancer, and others' poor outcomes interrupting positive approaches to their illness. Family and friends provided psychosocial and practical support, though living with family did not guarantee this. Furthermore, individual patients' personality, outlook on life, and behaviour, impacted on their desire to seek and receive support, and on the family's ability to provide it, with some patients choosing not to disclose their diagnosis. Our study also indicated that positive interactions with HCPs, notably nursing staff, appeared to be largely determined by the patient's pathway; the majority of those who considered nurses as emotionally supportive had received treatment. Finally, both patients and relatives discussed the impact of the CHC on family and friends, stressing the needs of these groups and highlighting gaps in current provision.

For peer review only - http://bmjopen.bmj.com/site/about/guidelines.xhtml

The unique nature of CHCs likely impacts upon patients' experiences of psychosocial support. For example, patients who did not disclose their diagnosis tended to be those with the most indolent disease, who were being managed on observation, and/or who felt their cancer was not impacting their life and was invisible to others. This lack of disclosure may not have been possible for patients with cancers that are more visible (e.g. symptomatic and/or requiring immediate or aggressive treatment). Accordingly, some patients highlighted reluctance to help family and friends understand why treatment was not currently necessary, in contrast to expectations following a cancer diagnosis. In addition, unlike many potentially curable cancers, relapse and further intervention is always possible some CHCs, and for diagnoses, is expected. Therefore, the emotional impact of living with a CHC is likely to vary and fluctuate over time, particularly given patients' increasing life expectancy. Thus ongoing, yet potentially intermittent support, which is responsive to the patient's position on their relapsing-remitting pathway, and their associated needs, is especially important.

Our findings are consistent with previous research observing barriers to support group attendance, and thus opportunities for social support. For example, Swash et al (2018) describe patient difficulties in remaining positive when others with the same diagnosis become increasingly unwell, or die (18). A reliance on family and friends for social and emotional support has also been reported (26,27,37). Other research discusses patients withholding cancer related anxiety and distress from friends and relatives to protect them from worry (13). Generic (e.g. Macmillan) cancer support services were rarely mentioned by interviewees in our study and some deemed these inappropriate for CHC. This echoes previous research on CHCs, in which participants didn't always identify themselves as cancer patients, largely due to their prolonged pathway, lack of obvious symptoms, and associated terminology (e.g. haematology not oncology) (18). Limited HCP contact during observation has also been highlighted, with hospital appointments occurring at lengthy (e.g. 3-12 month) intervals, thus reducing opportunities for emotional support (18,23). Resonating with our study, some patients feel uncomfortable raising emotional concerns with HCPs, and feel HCPs do not want to discuss such issues, thereby enhancing barriers to support (18). The Serious Illness Care Program (SICP) may be a useful approach to improving communication between patients, families and HCPs (38). Initially designed as a palliative care intervention, recent research highlights the benefits of introducing SICP earlier in CHC patient journeys to encourage dialogue and enable discussion about fears and concerns associated with the cancer, beyond those related to end of life (39).

The recent National Cancer Patient Experience Survey (NCPES) found 92.8% of patients with blood cancer said they had a main contact, most often a nurse, to support them during treatment, with most finding these individuals reliable and helpful (40). HCPs are also in a position to recommend and increase awareness of sources of support (41), with the NCPES reporting that 89.1% of patients with blood cancers felt hospital staff provided guidance on support groups and other resources, and 78.4% indicating they received the right amount of support with their health and wellbeing. However, only 29.1% of those who felt they needed emotional support could access this at home post-treatment (40). This was slightly lower than the overall score for all cancer patients (31.8%), but is likely to be particularly relevant to those with CHCs, where there is no cure and thus further monitoring, and possibly treatment, is needed. Whilst clinical staff appreciate that CHC patients' emotions may change over time (42), they may underestimate the impact of these diseases on individual lives and psychological wellbeing (15,43), and research is required to fully understand needs at various timepoints. Furthermore, clinical staff, including CNSs, may lack the confidence, time and resources to deal with the psychosocial consequences of cancer (19,41), or may use their time to focus on physical disease aspects (44,45), which could result in the under provision of emotional support (17).

For peer review only - http://bmjopen.bmj.com/site/about/guidelines.xhtml

Many sources of support discussed by patients are likely to have been impacted by the COVID-19 pandemic, due to lockdown restrictions and infection concerns, given that patients with haematological cancers are particularly vulnerable (46). This could result in some choosing to avoid face-to-face groups and services, or being encouraged to do so by HCPs. Online peer support was discussed by patients, and has been found to improve psychosocial wellbeing in cancer patients, but is recommended as complementary to face-to-face support rather than a replacement (47). Providing counselling or similar psychological support via videoconferencing has also been suggested, though barriers have been identified including: concerns regarding confidentiality, technical issues and software requirements (e.g. computer and webcam), and whether patients are able to talk openly in private, whilst in their own home (48,49).

Previous research has discussed the impact of cancer on patients' families and carers, including worsening psychological wellbeing, physical health and financial security (50–53), which may vary throughout illness (54). Suggestions to improve family outcomes include formally measuring caregiver needs, education and empowerment (55). A review of measures to assess the impact of caring found that few such tools had been systematically evaluated for cancer populations (56). There were also gaps, with a limited number covering changing family dynamics (which we found to be important), and many measures being outdated, thus potentially irrelevant, especially for people living longer with cancer (56). CHCs present specific problems due to their relapsing-remitting nature and the anxiety, repeated upheaval and on-going uncertainty for patients and families. The loneliness and confusion experienced by some family members in our study suggests that peer support would be useful. A recent feasibility study found one-to-one peer support was acceptable and beneficial for caregivers of those with newly diagnosed haematological cancers (57), with similar findings for group-based support (58).

This study has allowed us to capture a breadth of experiences and changing attitudes by interviewing patients at varying intervals post diagnosis, and with different treatment pathways. Although prolonged time since diagnosis may affect recall, referring back to diaries and calendars is likely to have mitigated this; moreover, in many cases, patient accounts were corroborated by relatives. Relatives were not present at every interview, a study limitation, especially with regard to views about support for carers and relatives. Our findings are likely to be transferable within the UK, and in countries with similar approaches to healthcare. However, we note that our sample only includes those over 50 years of age, and whilst the majority of CHCs are diagnosed in this population, the concerns of younger adults with CHCs may not have been identified. This should be considered in future research, as concerns among this group are likely to differ, possibly including greater emphasis on the potential impact of the CHC on fertility and family life (59), or the potential for symptoms and/or treatment side effects to impact patients' professional lives, either due to needing to retire or having to adapt their working patterns (37,60,61).

Conclusions

Individual preferences and attitudes resulted in marked differences concerning perceived needs for support, whether and how this was accessed, and the sources utilised. Relationships with others, including HCPs and family members, influenced whether support was considered to be available and if patients drew upon it. Preferences were also found to vary within individuals over time, mainly due to the long-term, changing nature of CHCs; the need for treatment after observation often leading patients to seek/receive additional support. Patients on observation may have specific difficulties accessing psychosocial support, due to decisions about diagnostic disclosure and limited HCP interactions, which should be considered if such patients are not to be overlooked. Unmet

For peer review only - http://bmjopen.bmj.com/site/about/guidelines.xhtml

needs were also evident, especially at key points of the clinical pathway, such as diagnosis and relapse, but also more generally, over time. Patients with CHCs may live with distress related to their condition for many years; thus, monitoring emotional needs is of equal importance as physical symptoms, particularly as the latter may never occur. HCPs are ideally placed for this role, as well as appropriate onward sign-posting/referral; although restricted time and resources may necessitate consideration of alternative approaches. Our findings underscore the need for patients with CHC to receive support that encompasses their psychosocial needs, and considers their unique treatment pathways and individual lives.

Figure 1. Diagram of the impact of patients' individual approaches on identified themes

For peer review only

Acknowledgements

The authors are grateful to the patients and relatives who participated in interviews and shared their experiences with us so openly.

Competing interests

None declared.

Funding

This work was supported by Cancer Research UK [29685], the National Institute for Health Research [RP-PG-0613-2002], and Blood Cancer UK [15037]. No funders were involved in the study design, data collection, analysis, interpretation or reporting.

Ethics approval

Details concerning HMRN's configuration, methods and ethical approvals can be found elsewhere (30). For the present study, ethical approval was provided by the London, City and East Research Ethics Committee (REC:16/ LO/0740).

Patient consent for publication

Not applicable.

Data availability statement

The datasets analysed during the current study are not publicly available as participants did not provide permission for their entire interview transcripts to be published, and ethical approval was not sought for this. A document containing the qualitative themes, and example codes and quotes is available from the authors on reasonable request.

For peer review only - http://bmjopen.bmj.com/site/about/guidelines.xhtml

Author contributions

DH, ER, AS and RP designed the study. AS identified potential participants. DM recruited participants and conducted interviews. Transcripts were coded and analysed by RS with input from DM, DH and AH. RS wrote the first draft of the manuscript, which was revised by DH, DM, AH, ER, AS and RP, who also commented on clinical aspects of the study. All authors read and approved the final manuscript.

For peer review only

For peer review only - http://bmjopen.bmj.com/site/about/guidelines.xhtml

REFERENCES

1. Swerdlow S, Campo E, Harris N, Jaffe E, Pileri S, Stein H, et al. WHO Classification of Tumours of Haematopoietic and Lymphoid Tissues. Lyon, France: World Health Organisation; 2017.

2. Sung H, Ferlay J, Siegel RL, Laversanne M, Soerjomataram I, Jemal A, et al. Global Cancer Statistics 2020: GLOBOCAN Estimates of Incidence and Mortality Worldwide for 36 Cancers in 185 Countries. CA: A Cancer Journal for Clinicians. 2021;71:209–49.

3. Office for National Statistics. Cancer registration statistics, England, 2017. Available from: https://www.ons.gov.uk/peoplepopulationandcommunity/healthandsocialcare/conditionsanddiseases/datasets/cancerregistrationstatisticscancerregistrationstatisticsengland (Accessed: June 2023)

4. Haematological Malignancy Research Network. Statistics. 2022. Available from: https://hmrn.org/statistics/incidence (Accessed: June 2023)

5. Howell DA, McCaughan D, Smith AG, Patmore R, Roman E. Incurable but treatable: Understanding, uncertainty and impact in chronic blood cancers—A qualitative study from the UK's Haematological Malignancy Research Network. PLOS ONE. 2022;10;17(2):e0263672.

6. Blood Cancer UK. Watch and wait. 2022. Available from: https://bloodcancer.org.uk/understanding-blood-cancer/watch-wait/ (Accessed: June 2023)

7. Bewarder M, Stilgenbauer S, Thurner L, Kaddu-Mulindwa D. Current Treatment Options in CLL. Cancers. 2021;13(10):2468.

8. Matasar MJ, Luminari S, Barr PM, Barta SK, Danilov AV, Hill BT, et al. Follicular Lymphoma: Recent and Emerging Therapies, Treatment Strategies, and Remaining Unmet Needs. Oncologist. 2019;24(11):e1236–50.

9. Rajkumar SV. Multiple myeloma: Every year a new standard? Hematol Oncol. 2019;37 Suppl 1:62–5.

10. Allart-Vorelli P, Porro B, Baguet F, Michel A, Cousson-Gélie F. Haematological cancer and quality of life: a systematic literature review. Blood Cancer Journal. 2015;5(4):e305.

11. Goswami P, Oliva EN, Ionova T, Else R, Kell J, Fielding AK, et al. Quality-of-life issues and symptoms reported by patients living with haematological malignancy: a qualitative study. Therapeutic Advances in Hematology. 2020;11:2040620720955002.

12. Waweru C, Kaur S, Sharma S, Mishra N. Health-related quality of life and economic burden of chronic lymphocytic leukemia in the era of novel targeted agents. Current Medical Research and Opinion. 2020;36(9):1481–95.

13. Maher K, Vries KD. An exploration of the lived experiences of individuals with relapsed Multiple Myeloma. European Journal of Cancer Care. 2011;20(2):267–75.

For peer review only - http://bmjopen.bmj.com/site/about/guidelines.xhtml

14. Molassiotis A, Wilson B, Blair S, Howe T, Cavet J. Unmet supportive care needs, psychological well-being and quality of life in patients living with multiple myeloma and their partners. Psychooncology. 2011;20(1):88–97.

15. Evans J, Ziebland S, Pettitt AR. Incurable, invisible and inconclusive: watchful waiting for chronic lymphocytic leukaemia and implications for doctor-patient communication. European Journal of Cancer Care. 2012;21(1):67–77.

16. Lamers J, Hartmann M, Goldschmidt H, Brechtel A, Hillengass J, Herzog W. Psychosocial support in patients with multiple myeloma at time of diagnosis: who wants what? Psycho-Oncology. 2013;22:2313-2320

17. Swash B, Hulbert-Williams N, Bramwell R. Unmet psychosocial needs in haematological cancer: a systematic review. Supportive Care in Cancer. 2014;22(4):1131–41.

18. Swash B, Hulbert-Williams N, Bramwell R. 'Haematological cancers, they're a funny bunch': A qualitative study of non-Hodgkin's lymphoma patient experiences of unmet supportive care needs. Journal of Health Psychology. 2018;28;1464–75.

19. Hulin C, Hansen T, Heron L, Pughe R, Streetly M, Plate A, et al. Living with the burden of relapse in multiple myeloma from the patient and physician perspective. Leukemia Research. 2017;59:75–84.

20. Tsatsou I, Konstantinidis T, Kalemikerakis I, Adamakidou T, Vlachou E, Govina O. Unmet Supportive Care Needs of Patients with Hematological Malignancies: A Systematic Review. Asia Pacific Journal of Oncology Nursing. 2020;8(1):5–17.

21. LeBlanc MR, LeBlanc TW, Bryant AL, Pollak KI, Bailey DE, Smith SK. A qualitative study of the experiences of living my multiple myeloma. Oncology Nursing Forum. 2021;48(2):151–60.

22. Rubins Z, Gibson BJ, Chantry A. What Can Patient Narratives Reveal to Us About the Experience of a Diagnosis of Myeloma? A Qualitative Scoping Review. Journal of Patient Experience. 2022;9:23743735221079132.

23. Damen MDC, Westerweel PE, Levin MD, Pelle AJ. Unmet supportive care needs, anxiety and depression in haematology patients during watch-and-wait. Psycho-Oncology. 2022;31(2):176–84.

24. El-Jawahri AR, Vandusen HB, Traeger LN, Fishbein JN, Keenan T, Gallagher ER, et al. Quality of life and mood predict posttraumatic stress disorder after hematopoietic stem cell transplantation. Cancer. 2016;122(5):806–12.

25. Haematological Malignancy Research Network. Survival. 2022. Available from: https://hmrn.org/statistics/survival (Accessed: June 2023)

26. Cuffe CH, Quirke MB, McCabe C. Patients' experiences of living with multiple myeloma. British Journal of Nursing. 2020;29(2):103–10.

27. Raphael D, Frey R, Gott M. Maintaining psychosocial wellbeing for post-treatment haematological cancer survivors: Strategies and potential barriers. European Journal of Oncology Nursing. 2019;38:36–41.

For peer review only - http://bmjopen.bmj.com/site/about/guidelines.xhtml

28. Tong A, Sainsbury P, Craig J. Consolidated criteria for reporting qualitative research (COREQ): a 32-item checklist for interviews and focus groups. International Journal for Quality in Health Care. 2007;19(6):349–57.

29. Haematological Malignancy Research Network. 2022. Available from: https://www.hmrn.org (Accessed: June 2023)

30. Roman E, Kane E, Howell D, Lamb M, Bagguley T, Crouch S, et al. Cohort Profile Update: The Haematological Malignancy Research Network (HMRN)'s UK population-based cohorts. International Journal of Epidemiology. 2022;e87–94.

31. Patton MQ. Qualitative Research and Evaluation Methods: Integrating Theory and Practice. Fourth Edition. Thousand Oaks, CA: Sage Publications; 2015.

32. Bowen GA. Naturalistic inquiry and the saturation concept: a research note. Qualitative Research. 2008;8(1):137–52.

33. Atherton K, Young B, Salmon P. Understanding the information needs of people with haematological cancers. A meta-ethnography of quantitative and qualitative research. European Journal of Cancer Care. 2017;26(6).

34. Atherton K, Young B, Kalakonda N, Salmon P. Perspectives of patients with haematological cancer on how clinicians meet their information needs: 'managing' information versus 'giving' it. Psychooncology. 2018;27(7):1719–26.

35. Braun V, Clarke V. One size fits all? What counts as quality practice in (reflexive) thematic analysis? Qualitative Research in Psychology. 2021;18(3):328–52.

36. Braun V, Clarke V. Conceptual and design thinking for thematic analysis. Qualitative Psychology. 2022;9(1):3–26.

37. Molassiotis A, Wilson B, Blair S, Howe T, Cavet J. Living with multiple myeloma: experiences of patients and their informal caregivers. Supportive Care in Cancer. 2011;19(1):101–11.

38. Bernacki R, Hutchings M, Vick J, Smith G, Paladino J, Lipsitz S, et al. Development of the Serious Illness Care Program: a randomised controlled trial of a palliative care communication intervention. BMJ Open. 2015;5(10):e009032.

39. Borregaard Myrhøj C, Novrup Clemmensen S, Sax Røgind S, Jarden M, Toudal Viftrup D. Serious illness conversations in patients with multiple myeloma and their family caregivers—A qualitative interview study. European Journal of Cancer Care. 2022;31(1):e13537.

40. NHS England. National Cancer Patient Experience Survey. 2021. Available from: https://www.ncpes.co.uk/2021-national-results/ (Accessed: June 2023)

41. Newton JC, O'Connor M, Saunders C, Moorin R, Ali S, Nowak AK, et al. The role of psychosocial support in the experiences of people living with advanced cancer: A qualitative exploration of patients' perspectives. Psycho-Oncology. 2021;30(3):287–95.

42. Liptrott SJ, Bee P, Lovell K. Providing Telephone-Based Support for Patients With Haematological Malignancies: A Qualitative Investigation of Expert Nurses. Canadian Journal of Nursing Research. 2022;54(2):121–33.

For peer review only

For peer review only - http://bmjopen.bmj.com/site/about/guidelines.xhtml

43. Hewison A, Roman E, Smith A, McCaughan D, Sheridan R, Patmore R, et al. Chronic myeloid leukemia: A qualitative interview study exploring disease impact from patient and practitioner perspectives. In Review. Available from: https://www.researchsquare.com/article/rs-2065284/v1 (Accessed: June 2023)

44. NHS National Cancer Programme. Excellence in Cancer Care: The Contribution of the Clinical Nurse Specialist. 2010. Available from: https://www.macmillan.org.uk/documents/aboutus/commissioners/excellenceincancercarethecontributionoftheclinicalnursespecialist.pdf (Accessed: June 2023)

45. Independent Cancer Taskforce. Achieving World-Class Cancer Outcomes - A Strategy for England 2015-2020. 2015. Available from: https://cancervanguard.nhs.uk/national-cancer-strategy/ (Accessed: June 2023)

46. Zomerdijk N, Jongenelis M, Yuen E, Turner J, Huntley K, Smith A, et al. Experiences and needs of people with haematological cancers during the COVID-19 pandemic: A qualitative study. Psycho-Oncology. 2022;31(3):416–24.

47. Allison KR, Patterson P, Guilbert D, Noke M, Husson O. Logging On, Reaching Out, and Getting By: A Review of Self-reported Psychosocial Impacts of Online Peer Support for People Impacted by Cancer. Proceedings of the ACM on Human-Computer Interaction. 2021;5(CSCW1):95:1-95:35.

48. Siegel A, Zuo Y, Moghaddamcharkari N, McIntyre RS, Rosenblat JD. Barriers, benefits and interventions for improving the delivery of telemental health services during the coronavirus disease 2019 pandemic: a systematic review. Current Opinion in Psychiatry. 2021;34(4):434–43.

49. Connolly SL, Miller CJ, Lindsay JA, Bauer MS. A systematic review of providers' attitudes toward telemental health via videoconferencing. Clinical Psychology: Science and Practice. 2020;27(2):e12311.

50. Girgis A, Lambert S, Johnson C, Waller A, Currow D. Physical, Psychosocial, Relationship, and Economic Burden of Caring for People With Cancer: A Review. Journal of Oncology Practice. 2013;9(4):197–202.

51. Pitceathly C, Maguire P. The psychological impact of cancer on patients' partners and other key relatives: a review. European Journal of Cancer. 2003;39(11):1517–24.

52. Williams AL. Psychosocial burden of family caregivers to adults with cancer. Psycho-Oncology. 2013;73–85.

53. Geng HM, Chuang DM, Yang F, Yang Y, Liu WM, Liu LH, et al. Prevalence and determinants of depression in caregivers of cancer patients. Medicine. 2018;97(39):e11863.

54. Kim Y, Given BA. Quality of life of family caregivers of cancer survivors. Cancer. 2008;112(S11):2556–68.

55. Berry LL, Dalwadi SM, Jacobson JO. Supporting the supporters: What family caregivers need to care for a loved one with cancer. Journal of Oncology Practice. 2016;13(1):8.

56. Shilling V, Matthews L, Jenkins V, Fallowfield L. Patient-reported outcome measures for cancer caregivers: a systematic review. Quality of Life Research. 2016;25(8):1859–76.

For peer review only

57. Husted Nielsen I, Piil K, Tolver A, Grønbæk K, Kjeldsen L, Jarden M. Family caregiver ambassador support for caregivers of patients with newly diagnosed hematological cancer: a feasibility study. Supportive Care in Cancer. 2022;30(8):6923–35.

58. Pailler ME, Johnson TM, Zevon MA, Kuszczak S, Griffiths E, Thompson J, et al. Acceptability, Feasibility, and Efficacy of a Supportive Group Intervention for Caregivers of Newly Diagnosed Leukemia Patients. Journal of Psychosocial Oncology. 2015;33(2):163–77.

59. Devine H, Verina D. Young Adults with Multiple Myeloma. Seminars in Oncology Nursing. 2017;33(3):316–31.

60. Potrata B, Cavet J, Blair S, Howe T, Molassiotis A. 'Like a sieve': an exploratory study on cognitive impairments in patients with multiple myeloma. European Journal of Cancer Care. 2010;19(6):721–8.

61. Forbat L, Sedgley T, Bellussi L. Multiple Myeloma in People of Working Age: A Systematic Review and Evidence Synthesis of Experiences of Paid and Unpaid Work. European Journal of Cancer Care. 2023;e6292479.

For peer review only

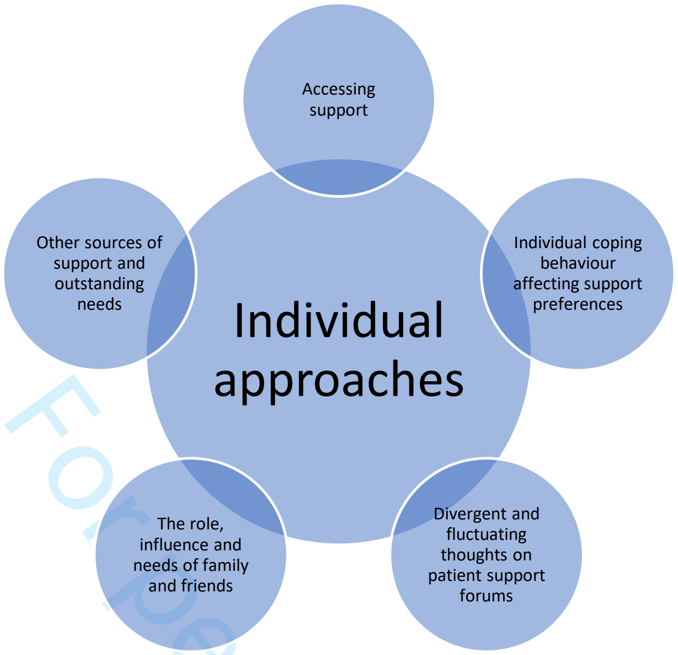

Figure 1. Diagram of the impact of patients' individual approaches on identified themes

Supplementary file 1
Details of sample

ID	Diagnosis[1]	Age range at interview (years)	Years since diagnosis	Known treatment line(s) preceeding interview[2,3]					
				1	2	3	4	5	6
P1	CLL	60-70	4	Observation	-	-	-	-	-
P2	MZL	60-70	15	Observation	Chemotx	Observation	-	-	-
P3	CLL	60-70	22	Observation	Chemotx	Observation	-	-	-
P4	MZL	60-70	3	Observation	Chemotx	-	-	-	-
P5	MZL	50-60	2	HPE	Observation	-	-	-	-
P6*	CLL	70-80	8	Observation	Chemotx	Observation	-	-	-
P7*	CLL	60-70	6	Observation	Chemotx	Observation	-	-	-
P8	FL	70-80	3	Chemotx	Radiotx	Observation	-	-	-
P9	CLL	80-90	5	Observation	Chemotx	-	-	-	-
P10	FL	70-80	8	Observation	Chemotx	Chemotx	Chemotx	-	-
P11	Myeloma	60-70	10	Observation	Chemotx	Observation	-	-	-
P12	MZL	70-80	5	Observation	Chemotx	-	-	-	-
P13	CLL	50-60	1	Observation	-	-	-	-	-
P14	Myeloma	60-70	4	Steroids	Radiotx	Chemotx	Chemotx	Chemotx	SCT
P15	FL	70-80	3	Observation	Chemotx	-	-	-	-
P16	Myeloma	60-70	2	Chemotx	Chemotx	Chemotx	SCT	Observation	-
P17*	FL	60-70	3	Observation	-	-	-	-	-
P18	Myeloma	60-70	3	Chemotx	Chemotx	Chemotx	SCT	Observation	
P19	FL	50-60	3	Steroids	Chemotx	Chemotx	Observation	-	-
P20*	CLL	70-80	4	Observation	-	-	-	-	-
P21*	Myeloma	70-80	3	Steroids	Chemotx	Chemotx	Chemotx	SCT	-
P22*	CLL	70-80	3	Observation	Clinical trial	Observation	-	-	-
P23	Myeloma	60-70	3	Observation	-	-	-	-	-
P24	FL	50-60	4	Steroids	Chemotx	Radiotx	Observation	-	-
P25	FL	60-70	4	Chemotx	Chemotx	-	-	-	-
P26	Myeloma	70-80	4	Observation	-	-	-	-	-
P27*	CLL	70-80	4	Chemotx	Observation	-	-	-	-
P28	Myeloma	60-70	4	Steroids	Chemotx	Chemotx	SCT	Clinical trial	Chemotx
P29	CLL	70-80	3	Clinical trial	Observation	-	-	-	-
P30*	Myeloma	70-80	2	Observation	-	-	-	-	-
P31*	Myeloma	70-80	2	Radiotx	Steroids	Chemotx	Observation	-	-
P32*	MZL	60-70	2	Observation	Chemotx	Observation	-	-	-
P33	Myeloma	50-60	3	Chemotx	Chemotx	SCH	Observation	-	-
P34	FL	50-60	4	Steroids	Chemotx	Chemotx	Chemotx	-	-
P35	Myeloma	50-60	2	Chemotx	Chemotx	Chemotx	Chemotx	SCT	Observation

[1] CLL – Chronic Lymphocytic Leukaemia; FL – Follicular Lymphoma; MZL – Systemic Marginal Zone Lymphoma.
[2] Chemotx = Chemotherapy; HPE = H. Pylori eradication; Radiotx = Radiotherapy; SCT = Stem cell transplant (all autografts); SCH = Stem cell harvest (shown as SCT did not take place).
[3] Does not include supportive care (e.g. blood product transfusions, plasma exchange, bisphosphonates, cell mobilization products)
*Relative present at interview

For peer review only

For peer review only - http://bmjopen.bmj.com/site/about/guidelines.xhtml

Supplementary file 2

Topic guide

(Tested with two patients prior to use in interviews)

Patients and relatives were asked to focus on key time-points (diagnosis, W&W, treatment), with respect to the topics included in the following three areas:

1) Understanding and impact of chronic blood cancer

- Looking back, what did you know about blood cancers, and your diagnosis specifically?
- What led you to your diagnosis initially? *(symptoms, suspected cancer?)*
- How important is it to you that you receive information about your cancer? *(why is that?)*
- How do you feel about the information given to you at diagnosis/start of treatment?
- What did you understand about your diagnosis? *(and others?)*
- What were your expectations of what would happen after diagnosis? *(treatment, follow-up)*
- What issues did you discuss at the time of your diagnosis? *(disease type, likely pathway, outcomes?)*
- What did you understand about your likely pathway and outcomes? *(immediate and later?)*
- What do you understand about your treatment?
- How do you feel about the future? *(optimistic, pessimistic?)*
- What impact has having a chronic blood cancer had on you? (activities, work, psychosocial issues)
- What strategies have you used to help you cope with your blood cancer?

2) Information

- How do you feel about getting information from HCPs generally? *(time constraints; use of language/terminology)*
- Do you feel the information given applies specifically to you? *(personalized, tailored, specific)*
- How healthcare practitioners (HCPs) ascertain your information needs?
- Is the information you received explained in a way you can understand? *(technical language; level of detail)*
- What do HCPs do to check if you understand the information they give you?
- How do you feel about asking questions? Are your questions always answered?
- Do you feel that your information needs are usually met? What worked well and could have been better? *(diagnosis; treatment initiation/cessation - examples)*
- What do you think about the timing of information from HCPs? When is the right time? *(at diagnosis; during clinic appointments; when disease status changes; at other times)*
- How do/did you feel about discussing the risks/benefits of different treatments with HCPs?
- How do you feel about discussing prognosis? *("a statement about expectations that refers to the likely course of the cancer and/or outcome") (want to know/not; timing; language)*
- What strategies do you use to absorb information? (*in general, how bad news is processed*)

3) Treatment decisions

- How do you feel about being involved in decisions with HCPs about your treatment?
- Have you been asked you if you want to be involved in decisions about treatment?
- Do you want to be involved in decisions? *(preference for patient only; clinician only; patient/clinician)*
- What should be considered during treatment decision making? *(effectiveness of treatment; side effects; prognosis; patient goals, values, preferences; impact on quality of life)*
- What might make it easier or harder for you to be involved in making decisions about your treatment? *(time; style of communication; how information is conveyed; explanations)*
- Are there particular time-points when it is harder to be involved in making decisions about treatment? *(diagnosis; treatment initiation/change; treatment cessation)*

For peer review only

COREQ (COnsolidated criteria for REporting Qualitative research) Checklist

No. Item	Guide questions/description	Page	Further detail
**Domain 1: Research team and reflexivity**			
**Personal characteristics**			
1. Inter viewer/facilitator	Which author/s conducted the interview or focus group?	5	DM
2. Credentials	What were the researcher's credentials? E.g. PhD, MD	n/a	BA, RN, MSc
3. Occupation	What was their occupation at the time of the study?	n/a	Senior Research Fellow
4. Gender	Was the researcher male or female?	5	Female
5. Experience and training	What experience or training did the researcher have?	n/a	Registered Nurse, conducted many qualitative interviews.
**Relationship with participants**			
6. Relationship established	Was a relationship established prior to study commencement?	n/a	No
7. Participant knowledge of the interviewer	What did the participants know about the researcher? e.g. personal goals, reasons for doing the research	n/a	No knowledge.
8. Interviewer characteristics	What characteristics were reported about the interviewer/facilitator? e.g. Bias, assumptions, reasons and interests in the research topic	n/a	Experienced qualitative health services researcher.
**Domain 2: Study design**			
**Theoretical framework**			
9. Methodological orientation and Theory	What methodological orientation was stated to underpin the study? e.g. grounded theory, discourse analysis, ethnography, phenomenology, content analysis	5	Reflexive thematic analysis.
**Participant selection**			
10. Sampling	How were participants selected? e.g. purposive, convenience, consecutive, snowball	5	Purposive.
11. Method of approach	How were participants approached? e.g. face-to-face, telephone, mail, email	5	Mail, followed up by telephone if expressed interest.
12. Sample size	How many participants were in the study?	5	35
13. Non-participation	How many people refused to participate or dropped out? Reasons?	n/a	None dropped out. 20 patients did not respond to invite for reasons unknown.
**Setting**			
14. Setting of data collection	Where was the data collected? e.g. home, clinic, workplace	5	Home, University.
15. Presence of non-participants	Was anyone else present besides the participants and researchers?	5	Relatives were present at 10 interviews.
16. Description of sample	What are the important characteristics of the sample? e.g. demographic data, date	5	Also found in Supplementary file 2.
**Data collection**			

For peer review only

17. Interview guide	Were questions, prompts, guides provided by the authors? Was it pilot tested?	5	Topic guide found in Supplementary file 1.
18. Repeat interviews	Were repeat inter views carried out? If yes, how many?	n/a	No repeat interviews were carried out.
19. Audio/visual recording	Did the research use audio or visual recording to collect the data?	5	Audio.
20. Field notes	Were field notes made during and/or after the interview or focus group?	n/a	No.
21. Duration	What was the duration of the inter views or focus group?	5	60-90 minutes
22. Data saturation	Was data saturation discussed?	n/a	Yes, interviews were continued until saturation.
23. Transcripts returned	Were transcripts returned to participants for comment and/or correction?	n/a	No transcripts were returned.
**Data analysis**			
24. Number of data coders	How many data coders coded the data?	5	RS, DM, DH, AH
25. Description of the coding tree	Did authors provide a description of the coding tree?	Figure 1	
26. Derivation of themes	Were themes identified in advance or derived from the data	F5	Data manged in Microsoft Word.
27. Software	What software, if applicable, was used to manage the data	n/a	No software was used.
28. Participant checking	Did participants provide feedback on findings?	n/a	No.
**Reporting**			
29. Quotations presented	Were participant quotations presented to illustrate the themes/findings? Was each quotation identified? e.g. participant number	6-11	Quotations identified by participant number.
30. Data and findings consistent	Was there consistency between the data presented and the findings?	6-11	Yes.
31. Clarity of major themes	Were major themes clearly presented in the findings?	6-11, Figure 1	Major themes were presented alongside subthemes.
32. Clarity of minor themes	Is there a description of diverse cases or discussion of minor themes?	6-11	Yes, a range of responses are considered.

Developed from: Tong A, Sainsbury P, Craig J. Consolidated criteria for reporting qualitative research (COREQ): a 32-item checklist for interviews and focus groups. *International Journal for Quality in Health Care*. 2007. Volume 19, Number 6: pp. 349 – 357

For peer review only

Experiences and preferences for psychosocial support: A qualitative study exploring the views of patients with chronic haematological cancers

Journal:	*BMJ Open*
Manuscript ID	bmjopen-2022-070467.R2
Article Type:	Original research
Date Submitted by the Author:	01-Aug-2023
Complete List of Authors:	Sheridan, Rebecca; University of York, Health Sciences<br>McCaughan, Dorothy; University of York, Health Sciences<br>Hewison, Ann; University of York, Health Sciences<br>Roman, Eve; University of York, Health Sciences<br>Smith, Alexandra; University of York, Health Sciences<br>Patmore, Russell; Castle Hill Hospital<br>Howell, Debra; University of York, Health Sciences
**Primary Subject Heading**:	Haematology (incl blood transfusion)
Secondary Subject Heading:	Health services research, Qualitative research
Keywords:	Leukaemia < HAEMATOLOGY, Lymphoma < HAEMATOLOGY, Myeloma < HAEMATOLOGY

I, the Submitting Author has the right to grant and does grant on behalf of all authors of the Work (as defined in the below author licence), an exclusive licence and/or a non-exclusive licence for contributions from authors who are: i) UK Crown employees; ii) where BMJ has agreed a CC-BY licence shall apply, and/or iii) in accordance with the terms applicable for US Federal Government officers or employees acting as part of their official duties; on a worldwide, perpetual, irrevocable, royalty-free basis to BMJ Publishing Group Ltd ("BMJ") its licensees and where the relevant Journal is co-owned by BMJ to the co-owners of the Journal, to publish the Work in this journal and any other BMJ products and to exploit all rights, as set out in our licence.

The Submitting Author accepts and understands that any supply made under these terms is made by BMJ to the Submitting Author unless you are acting as an employee on behalf of your employer or a postgraduate student of an affiliated institution which is paying any applicable article publishing charge ("APC") for Open Access articles. Where the Submitting Author wishes to make the Work available on an Open Access basis (and intends to pay the relevant APC), the terms of reuse of such Open Access shall be governed by a Creative Commons licence – details of these licences and which Creative Commons licence will apply to this Work are set out in our licence referred to above.

Other than as permitted in any relevant BMJ Author's Self Archiving Policies, I confirm this Work has not been accepted for publication elsewhere, is not being considered for publication elsewhere and does not duplicate material already published. I confirm all authors consent to publication of this Work and authorise the granting of this licence.

For peer review only

Experiences and preferences for psychosocial support: A qualitative study exploring the views of patients with chronic haematological cancers

AUTHORS: Rebecca Sheridan, Dorothy McCaughan, Ann Hewison, Eve Roman, Alexandra Smith, Russell Patmore, Debra Howell

CORRESPONDING AUTHOR:

Debra Howell
Epidemiology & Cancer Statistics Group, Department of Health Sciences, Seebohm Rowntree Building, University of York, York, UK. YO10 5DD.
Telephone: +44 (0) 1904 321895
Email: debra.howell@york.ac.uk

CO-AUTHORS

Rebecca Sheridan
Epidemiology & Cancer Statistics Group, Department of Health Sciences, Seebohm Rowntree Building, University of York, York, UK. YO10 5DD.
Telephone: +44 (0) 1904 321079
Email: rebecca.sheridan@york.ac.uk

Dorothy McCaughan
Epidemiology & Cancer Statistics Group, Department of Health Sciences, Seebohm Rowntree Building, University of York, York, UK. YO10 5DD.
Telephone: +44 (0) 1904 321348
Email: dorothy.mccaughan@york.ac.uk

Ann Hewison
Epidemiology & Cancer Statistics Group, Department of Health Sciences, Seebohm Rowntree Building, University of York, York, UK. YO10 5DD.
Telephone: +44 (0) 1904 321916
Email: ann.hewison@york.ac.uk

Eve Roman
Epidemiology & Cancer Statistics Group, Department of Health Sciences, Seebohm Rowntree Building, University of York, York, UK. YO10 5DD.
Telephone: +44 (0) 904 321882
Email: eve.roman@york.ac.uk

Alexandra Smith
Epidemiology & Cancer Statistics Group, Department of Health Sciences, Seebohm Rowntree Building, University of York, York, UK. YO10 5DD.
Telephone: +44 (0) 904 321884

For peer review only

For peer review only - http://bmjopen.bmj.com/site/about/guidelines.xhtml

Email: alexandra.smith@york.ac.uk

Russell Patmore
Queens Centre for Oncology and Haematology, Castle Hill Hospital, Cottingham, UK.
Telephone: +44 (0) 1482 604444
Email: russell.patmore@nhs.net

KEYWORDS: Haematological cancer; psychosocial; peer support; myeloma; leukaemia; lymphoma

WORD COUNT: 5662, including all quotations

For peer review only

ABSTRACT

Objectives Patients with chronic haematological cancers are often treated on a relapsing-remitting pathway, which may extend for many years. Such diagnoses are associated with uncertainties that often cause anxiety and distress, meaning patients (and families) are susceptible to potentially prolonged emotional difficulties, across the cancer journey. Experiences and preferences regarding psychosocial needs and support over time are relatively unexplored, which this study aimed to address.

Setting and design Set within the UK's Haematological Malignancy Research Network (HMRN: an ongoing population-based cohort that generates evidence to underpin improved clinical practice) a qualitative, exploratory study was conducted, utilising semi-structured interviews. Reflexive thematic analysis was used to assess the interview data via an exploratory, inductive approach, underpinned by the research questions.

Participants Thirty-five patients were included with chronic lymphocytic leukaemia, follicular lymphoma, marginal zone lymphoma, or myeloma; 10 of whom were interviewed alongside a relative.

Results Five themes were identified from the data: (i) accessing support; (ii) individual coping behaviour affecting support preferences; (iii) divergent and fluctuating thoughts on patient support forums; (iv) the role, influence and needs of family and friends; and (v) other sources of support and outstanding needs. Findings suggest that patients' individual attitudes towards support varied over time. This also influenced whether support was perceived to be available, and if it was then utilised.

Conclusion This study highlighted the variation in preferences towards psychosocial support among patients with chronic haematological cancers. As patients can live for many years with significant emotional difficulties, they may benefit from frequent monitoring of their psychosocial wellbeing, as well as signposting to holistic support, if this is needed.

For peer review only

ARTICLE SUMMARY

Strengths and limitations of this study

- Comprehensive findings due to inclusion of patients with a range of chronic haematological cancers, demographic characteristics and treatment pathways.
- Robust data collection and analysis conducted by an experienced team of qualitative researchers.
- Transferable findings within the UK and countries with similar healthcare systems.
- Potential recall difficulties due to time since diagnosis, despite the use of diaries/calendars.
- Limited inclusion of relatives/carers (due to patient preferences) and patients under 50 years of age (due to disease rarity); hence the need for further research with these groups.

For peer review only - http://bmjopen.bmj.com/site/about/guidelines.xhtml

INTRODUCTION

Haematological malignancies (blood cancers) are a diverse group with many sub-types (1). Broadly classified as leukaemias, lymphomas and myeloma, these are collectively the fifth most common cancer globally (2), with increased incidence amongst ageing populations (3,4). While some of these malignancies are potentially curable, the majority (around 60%) are not, in which case treatments aim to ameliorate symptoms and slow progression (5). The four most common chronic haematological cancers (CHCs) are chronic lymphocytic leukaemia (CLL), follicular lymphoma (FL), marginal zone lymphoma (MZL) and myeloma. Some patients with such conditions may be treated from diagnosis; while others will be managed on 'watch and wait', either never requiring treatment, or alternating between treatment and observation with the aim of restoring remission (6). Treatments may include combinations of chemotherapy, radiotherapy, stem cell transplant and targeted agents (7–9).

Patients with CHCs may be successfully managed on relapsing-remitting pathways over many years, though symptom burden and loss of functionality can cause increased dependence on others, and may have a detrimental effect on quality of life (10–12). Living with a CHC may be associated with increased emotional and psychological distress, linked primarily to uncertainty concerning disease progression/relapse, treatment, and prognosis, compounded by poor diagnostic understanding, and informational deficiencies (5,13–22). Accordingly, the most commonly reported unmet supportive care needs discussed by CHC patients are informational and psychological (23). The importance of timely psychosocial support during observation, and before, during, and after treatment is underscored in a prospective, longitudinal study, which notes an increased risk of patients developing psychological disorders when supportive needs are not met (24).

CHC patients' experiences of accessing and receiving support remain relatively unexplored, despite the growing significance of this issue due to improved outcomes requiring long-term follow-up; five-year relative survival estimates range from 48% (myeloma), to 88% (FL) (25). Relevant qualitative literature emphasises the value patients place on social support, which also extends to family and significant others, to enable adjustment and coping (13,18,26,27). Patients may also rely on peer support groups and health care professionals (HCPs), such as clinical nurse specialists (CNSs), for emotional as well as informational support (18,27).

Understanding patients' views of seeking and receiving support, and associated barriers and facilitators, is circumscribed by the small-scale of many published studies, predominantly focusing on patients with myeloma (13,14,19,21,26). Our own study therefore elicited in-depth perspectives from a comparatively large qualitative sample of thirty-five patients with CLL, FL, myeloma and MZL. Data were explored with the aim of examining the experiences of seeking and receiving emotional and psychological support, from the point of diagnosis onwards, and identifying perceived needs and preferences.

METHODS

This study is part of a broader programme of work examining the perspectives of CHC patients concerning information needs and treatment decisions (5). Methods and results are reported according to the Consolidated Criteria for Reporting Qualitative Research guidelines (28).

For peer review only - http://bmjopen.bmj.com/site/about/guidelines.xhtml

Study design and setting

A qualitative, exploratory study was conducted, using semi-structured interviews. It was set within the UK's Haematological Malignancy Research Network (HMRN: https://hmrn.org) (29); a population-based cohort that was established in 2004 across an area of 4 million people with similar socio-demographic characteristics to the UK as a whole (30). HMRN is an on-going collaboration between university academics, National Health Service (NHS) clinicians, and patients and carers, who collectively co-design and conduct research, with the aim of generating evidence that can be used to improve clinical practice, locally, nationally and internationally.

Sampling

Sampling was purposive; we aimed to capture a range of experiences relating to the topic of interest (31). Participants with CLL, FL, myeloma or MZL were selected according to the median diagnostic age for each cancer, with variation by gender, time since diagnosis, and events on the clinical pathway; an overview of patient characteristics can be found in Supplementary file 1. Refinement of the sampling strategy led to inclusion of patients across broader demographic categories; for example, by including those above and below sub-type median diagnostic age.

Data collection

Checks with patients' clinical teams ensured they were well enough to participate in the study. Identified individuals were sent information and the researcher's contact details to discuss participation. Interviews were conducted by an experienced female researcher (DM), between February-October 2019, at a time and place of the patient's choosing, usually their home, with a friend or relative present if desired. Interviews continued until data saturation had been reached, defined as the point at which no new insights could be added to the analysis (32). Participants were invited to ask questions about the study prior to interview, and assurances were given regarding anonymity and confidentiality. Written consent was obtained from all participants, including for the use of direct quotations. Interviews lasted 60-90 minutes, and were digitally audio-recorded. A semi-structured topic guide (Supplementary file 2), developed from the extant research literature (15–17,33,34), and in conjunction with haematology practitioners, was used flexibly to guide discussions, which were subsequently transcribed verbatim, and checked, corrected and pseudonymised by the interviewer.

Data analysis

Reflexive thematic analysis was used, an approach more akin to a transtheoretical technique, than a methodology predicated on a particular theoretical stance (35,36). Analysis was carried out by an experienced team, using an exploratory, inductive approach, underpinned by the research questions. Initial codes were developed by the first author (RS), with further coding and development of themes being a collaborative process involving RS, DM, DH and AH. This was based on active searching for patterns of meaning across the data set, achieved through the dual processes of immersion in the data, and distancing, to allow time for reflection and the development of insights. Regular meetings (RS, DM, DH, AH) focussing on theme review and refinement, supplemented by written memos and reflections, promoted critical thinking, and enhanced reflexivity and interpretive depth. The overall aim was to 'interrogate' the data to arrive at an interpretation that was *'insightful, thoughtful, rich…nuanced'* (36). Patient quotes are presented in italics and refer to participant numbers (e.g. P1 for the patient and P1R for the patient's relative).

For peer review only - http://bmjopen.bmj.com/site/about/guidelines.xhtml

Patient and public involvement

Patients and relatives who regularly attended a blood cancer support group were involved in prioritising the study aims, preparing the funding application and disseminating findings. Long-standing links between the research team and members of this group facilitate their active involvement in HMRN's activities, although these patients and relatives did not take part in the interviews.

RESULTS

Thirty-five patients participated in the study; ten relatives contributed to interviews. Twelve patients had myeloma, 10 CLL, 8 FL and 5 MZL; treatment pathways varied (Supplementary file 1). Twenty-two patients were aged ≥60 years; and 19 were male.

Five themes were identified: (i) accessing support; (ii) individual coping behaviour affecting support preferences; (iii) divergent and fluctuating thoughts on patient support forums; (iv) the role, influence and needs of family and friends; (v) other sources of support and outstanding needs. Individual preferences were evident in each theme, with the patient's personality impacting their use of support (Figure 1).

Theme 1: Accessing support

Support was available from a number of sources, but was impacted by the patient's diagnosis and awareness of the resources available, informed by their own research and/or HCP knowledge and advice.

Subtheme 1.1 Varied types of support

Patients reported accessing or awareness of various types of peer support, including face-to-face/ online forums, and being introduced to, or knowing, other patients who shared their diagnosis. The majority of face-to-face support groups were not disease specific, but accommodated people with any haematological cancer. Online cancer specific groups referenced included Myeloma UK, CLL Support Association and also Facebook groups.

Informal support from family and friends was particularly important, whilst nurses, and, sometimes doctors, also contributed. Patients utilised NHS and private counselling services and complementary therapies such as acupuncture. Macmillan centres were attended by some, as well as support days and events hosted by charities, such as Myeloma UK.

Subtheme 1.2 Knowing where to find support

Most patients knew about online and face-to-face support groups, even if they did not want to use these resources. It was not always clear how patients became aware of support groups; some were told by their doctor or nurse (P10, P12, P28), or received information via leaflets (P5). P19 and P3 specifically noted finding websites/forums based in America; P19 found *'it wasn't anything particularly helpful'* due to their non-UK focus.

Some patients contacted others who shared their diagnosis via their GP or family and friends. For example, P14's GP organised for someone with the same condition to speak to him: *'hearing from somebody who's been through it all and got the badge certainly helped'.* P33 had requested a

For peer review only - http://bmjopen.bmj.com/site/about/guidelines.xhtml

'buddy', something P21R would have welcomed for herself and her relative: *'someone that's like a year in to having this, who would sit down and explain what they discovered and how it works, and what to expect'.*

Some patients (P1, P4) requested counselling, while others (P9, P13) were offered a referral by nursing staff. Nurses also informed some patients (P8, P25) about other measures that might help, such as therapeutic massage. P16 had not been told about Macmillan support centres, but would have found this useful; it was unclear whether other patients were informed about them.

Subtheme 1.3 Impact of disease subtype on accessing peer support

A number of patients commented on difficulties due to their cancer subtype, such as its rarity (P5, P25, P33). Because of this, P25 felt *'on me [sic] own'* and unsupported, noting that neither her nor her daughter could find any support groups. This could be problematic even for patients who shared the same diagnosis; P5 explained people at her support group all had lymphoma or leukaemia but she didn't perceive herself as having the same disease due to differences in site, clarifying: *'I have [the same cancer] but it's in my stomach'.*

Theme 2: Individual coping behaviour affecting support preferences

The patient's personal approach to their cancer influenced their coping strategies, often resulting in a reluctance to access formal support.

Subtheme 2.1 Perceptions of illness: Distancing, distraction, fighting back and maintaining hope

A number of patients didn't want to *'dwell'* on their cancer and discussed measures taken to actively distance or distract themselves. Instead of seeking specific support, such individuals focused on other activities, with P17 saying: *'We have quite a lot of interests, so we're always doing things and I think that's good because it just takes your mind off things'.* P27 and P29 took a philosophical approach to their cancer: *'there's nothing you can do about it…you make the best…of what is available for you'* (P27). Patients did not want to be defined by their condition, or for their condition to *'rule [their] life'* (P3). P5 attended a support group infrequently, as they didn't want their cancer to *'represent'* them. Similarly, P35 commented: *'for some people [at support group] it's like their whole life is having myeloma. I just don't want to be like that'.*

Many patients highlighted specific coping strategies to distract themselves from their CHC, including exercising, dog walking and gardening. Having another focus was important to P3 when trying to accept their diagnosis and deferred treatment (watch and wait): *'[running] made a massive difference because I'm doing something…I'm fighting back'.*

Some people felt they needed to stay positive and maintain hope with respect to their cancer and future outcomes. P24 adopted a *'positive mental attitude';* P25 described focussing on future events as *'summit to aim for'.* P13 felt that support group attendance might interfere with their positive approach due to the sharing of negative experiences, explaining: *'I wouldn't want to hear all of the things, you know, "well, it did this to me and it did that to me, and I thought I was on death's door", …I need to keep buoyant and positive about it'.*

For peer review only - http://bmjopen.bmj.com/site/about/guidelines.xhtml

Subtheme 2.2 Perceptions of personality

Some patients felt they were not the 'type' to attend a support group: *'I am just a bit introverted meself [sic]…I'm not a kind of a support group type person…'* (P21). Similarly, P27 described such meetings as *'not for me'*, but did acknowledge that this could change in the future. Whilst P10 appreciated that support groups could be useful for others, they found them unnecessary due to them being inherently positive: *'I have a positive attitude and I don't feel I need support'.*

Theme 3: Divergent and fluctuating thoughts on patient support forums

Patients referred to both the significant benefits and also drawbacks of face-to-face and online support forums, with variations noted between individuals.

Subtheme 3.1 Varied perspectives

From a positive perspective, support groups were described as *'very valuable'* (P1); P6 described their group leader as their '*rock'.* Meeting others in a similar situation provided patients with reassurance and comradery: *'you're not on your own but to know somebody else is going through it, and you can tell her your worries'* (P3). P33 also noted that their efforts to maintain positivity within support groups had helped others, saying that members had remarked: *'you've cheered me up and made me feel positive because you're always so positive'.*

Whilst positive experiences were welcomed, hearing negative accounts could be frightening, reflecting patients' desires not to be exposed to these stories (see subtheme 2.1). For example, P35 explained: '*I thought I'd find [support group] terrifying, which I sort of do really because it's like people just talking about their terrible stories a lot of the time'.* P7 highlighted that patients may hear both positive and negative accounts, with the negative making them feel like they were '*in a queue sat there waiting to die'.*

Subtheme 3.2 Changing preferences over time

Patients commented on their decisions regarding when to seek group support, often depending on the status of their cancer. For example, some patients (P4, P8) did not attend face-to-face meetings during treatment due to fatigue, illness or infection risk. Engagement could increase when they needed information or advice, such as when making treatment decisions. As explained by P3, who became more active on internet forums at relapse: *'A lot of people were saying, yeah I was the same as you…I just wanted to forget about it but then when you have something to remind you that you've still got the condition a lot of people start to be more active'.* Some patients on observation didn't feel a support group was necessary as they were '*coping well'* (P17), or because their cancer didn't '*affect [their] lifestyle'* (P20).

Subtheme 3.3 Information provision

Patients generally found support groups and websites a useful source of information, and some noted that they themselves had provided information to others (P6, P13, P19). On websites in particular, patients could choose what information they wanted to engage with, and when. For example, P19 found negative material unwelcome saying: *'it used to scare me'.* P3 highlighted the importance of understanding that *'everyone is different'*, explaining: *'you have to decide what suits*

For peer review only - http://bmjopen.bmj.com/site/about/guidelines.xhtml

you, what you feel comfortable with and learn to take the advice that's useful for you and ignore the advice that's less useful'.

Theme 4: The role, influence and needs of family and friends

All patients discussed their family and friends, largely referring positively to the support these people provided. The impact of the patients' CHC on family and friends was also considered, the importance of which was recognised by patients.

Subtheme 4.1 Emotional and practical support: Reassurance, companionship and the importance of family and friends

Family and friends provided significant emotional support to the majority of patients; for example, P2 commented: *'the people who are close to you are very important because they can keep you going…I think they're very important in reassuring you'*. Patients also acknowledged the importance of practical support, including relatives or friends accompanying them to hospital appointments, reminding them what was discussed, and learning about their condition. P9 explained '*my daughter knows as much about my treatment as I do. She keeps really up-to-date with it and she's with me most treatments'.* Contrastingly, P3 attended appointments alone, because his wife would *'worry herself silly',* as appointments were *'more stress inducing'* for her than for himself. P34, who attended appointments alone due to his wife's work commitments, acknowledged that, with hindsight, practical support would have been useful.

Emotional and practical input from friends was particularly important for patients without immediate family support. P8, who lived alone, described her friend as '*marvellous',* explaining: *'I haven't got any family…She was just there for me the whole time'.* P33, whose partner had died, felt the need for practical and emotional support: *'I have to do all my own research, all my own ringing around and sometimes I think I just wish I had that person…I want somebody just to hold my hand and go, I'm going to sort that for you'.* For some, having family did not guarantee support, as exemplified by P25, who relied on a friend to provide emotional and practical help, despite living with relatives.

Subtheme 4.2 Disease characteristics and family dynamics: Impact of the cancer, perceived response of others and life context

Patients' decisions about disclosing their CHC impacted available support, and were often influenced by factors relating to their cancer. When patients felt their diagnosis would impact their lives, they often chose to tell family and friends. For example, P22 explained: *'we knew we were always going to always be at hospital…so we took the road that we would tell them'.* In contrast, when patients didn't feel their cancer was noticeably impacting them, for example when on observation, they did not always disclose their diagnosis, thus eliminating opportunities for support. P2 said: *'close family knew about it and close friends and that was it because I could get on with me [sic] life. I could manage it. Nobody needed to know'.* P20 also highlighted the nature of 'watch and wait', explaining they did not inform people about their diagnosis as: *'they wouldn't understand that there's no treatment required'.*

For peer review only - http://bmjopen.bmj.com/site/about/guidelines.xhtml

Patients hesitated to disclose their CHC to children for various reasons including their young age, because they were undertaking important exams, and/or because they did not wish to be '*a burden*' (P32). P35 knew she had to tell her children as her diagnosis was *'a big life changing event'* but was unsure about the timing, explaining: '*I just wanted to know if I could carry on my life without telling them'.* Being on watch and wait allowed P11 to keep their diagnosis from their children, who they believed were too young to understand/cope.

Even where family and friends were informed about the diagnosis, feelings were not always shared. For example, P7R explained that they did not discuss their worries in order to '*protect'* family. Similarly, P5 felt they had to be strong for others, so were providing, rather than receiving, support. Some patients did not share their feelings about their CHC even with close family, or were reluctant to ask for help, so could not be supported. For example, P2 described shock at their diagnosis, but his wife had said: *'the trouble was you didn't tell me all this so I couldn't be involved'.* P25 described feeling lonely, but wanted to *'make it easier for them [family]'*, admitting: *'I won't ask nobody for help. If somebody, if me [sic] cousin had offered to come, I would have said, "no I'm fine"'.*

Family dynamics could change as a result of the CHC diagnosis. P19 said: *'I didn't feel well and for the first time ever, my family just took full control of everything and normally I'm the one that's like the matriarch'.* However, whilst the patient was grateful, the diagnosis and changing circumstances were not accepted by everyone: *'[Daughter] wouldn't even talk about it and to this day she doesn't really. She just, you know, she just likes her mum to be mum, and functional'.*

Subtheme 4.3: The importance of support for family and carers

This was discussed by several patients, with P12 explaining they felt support was '*just as important for the family as it is for the patient'.* Patients appreciated that family are also impacted by their diagnosis; P11 suggested '*it's probably harder on my wife or my children'.* Similarly, P28 remarked that: '*The worry is done by the carers'.* In contrast, P6 acknowledged differences in perceptions of burden between the patient and their family: *'Mentally, [husband] forgets sometimes and he admits – "I forget you've got leukaemia". But it lives with me 24/7. I never forget that I've got it'.*

Patients' families found support in various settings. For example, P12 and P13 described nurses offering support to relatives, with P12 commenting that speaking to the nurse '*kind of settled [wife] a bit'.* P11 encountered another patient, also with myeloma, and explained how their wives then talked and found this useful: '*[Wife (of P11)] was a bit left in the dark she felt, so she really enjoyed talking to [contact's wife] about how she felt'.* Support was also available via dedicated Cancer Centres, with P18's partner accessing counselling and a therapeutic course. P28 praised Myeloma UK for their approach to supporting both patients and carers, including holding sessions specifically for carers at their events.

In contrast, some family members commented that their support needs were unmet. P7R described there being no information about support for relatives, saying '*it's lonely',* and highlighting that the carer also has a *'burden'.* P21R felt unsupported, suggesting counselling should be provided for patients and relatives. Sometimes support was available, but relatives were unwilling to accept it. For example, P19 commented that her daughter really needed support but wouldn't access it: *'the Macmillan nurse was really good and she offered to speak to [daughter], and she tried to ring her and she didn't want to talk about it'.*

For peer review only - http://bmjopen.bmj.com/site/about/guidelines.xhtml

Theme 5: Other sources of support and outstanding needs

Support from HCPs and other formal sources, such as counselling services, were discussed by some, with wide variation in the perceived usefulness of these resources. Unmet emotional needs were evident, however, particularly at significant time points in the patient's journey.

Subtheme 5.1 Perceptions of support from healthcare professionals

Nursing staff were considered a valuable source of support, often providing reassurance and encouragement for patients. For example, P14 said: '*[Nurse] has been there for me and guided me, cajoled me, pointed me in the right direction'.* There were differences in the type of support patients perceived nurses as providing; P5 described it as *'medical'* but '*not necessarily psychological'*, whilst P7 said: *'As well as your physical condition [nurses] were bothered about my emotional condition…'.*

A number of patients (P1, P16, P34) reported support from nurses as reliable and always available. In contrast, others were reluctant to access such input; P4 explained: *'I didn't feel I could be ringing [nurse] every day…because obviously she's got lots of other things to do and lots of other people to deal with'*. P4 acknowledged most nurses were *'understanding'*, but felt some trivialised her concerns: '*you'd ring up and they'd sort of brush it off'.* P4 and P33 expressed disappointment when nurses reneged on their stated intentions, including referral for counselling, and providing reassurance to family members.

Interactions with nursing staff appeared to vary according to CHC management, with patients on treatment (attending regularly) having greater contact than those being observed on watch and wait (attending less frequently). For example, a number of patients with myeloma (P11, P14, P18, P28), in frequent contact with nurses during treatment, discussed the importance of receiving regular support: '*we've had a lot of dealing with [nurses] and they're spot on'* (P11). Similarly, P3 said: *'The only time I can remember talking to nurses a lot was when I had my treatment and of course they were absolutely superb'.* Preferences were found to vary among those given contact details for nursing staff during periods of observation, with P2 choosing not to use these; while others appreciated this information and felt support was available if needed (P13, P17, P26). Contrastingly, P13 (solely observed since diagnosis) perceived nurses as providing '*holistics…mind, body and soul'* care to those on watch and wait, in order to *'free up the doctors, so they can see the patients that maybe need a little bit of extra time'*.

Whilst most comments about HCP support were about nurses, some patients referred to support from doctors, which was mainly, but not uniformly, perceived positively. P9 felt their doctor was *'very supportive and helpful'* when they were upset about needing chemotherapy, and P12 felt their doctor had '*plenty of empathy'.* In contrast, P4 described their doctors as speaking on a *'consultant level, with no emotions'.* A number of patients highlighted positive interactions with their GPs, with P30 describing theirs as *'fantastic'.* However, others (P6, P13, P23, P25) felt their GP lacked knowledge about their CHC. Three patients mentioned receiving support from other HCPs outwith the NHS, including nurses from Myeloma UK (P29) and Macmillan (P13, P19).

Subtheme 5.2 Counselling and complementary support

Seven patients discussed their experiences of counselling, with perceptions varying markedly. P1 felt this had been useful, facilitating the realisation that they had '*experienced a tremendous trauma'*, which had impacted their behaviour in response to treatment and infection fears. Conversely, P25 felt counselling was '*a complete waste of time'* and not what she had expected, explaining: *'To me, a*

For peer review only - http://bmjopen.bmj.com/site/about/guidelines.xhtml

counsellor is somebody that will counsel you and advise you. She never opened her mouth'. Nevertheless, P25 did feel the service had helped her '*come to terms*' with her diagnosis. P33 felt their psychologist only cared how they felt about their treatment, and not their wellbeing more generally. P35 described both positive and negative aspects of counselling, explaining that whilst it was '*helpful'* it could also be upsetting to discuss their feelings.

Four patients (P9, P16, P19, P22) referred to Macmillan services, all positively, though P22 felt these weren't aimed at her as she wasn't *'terminal'*. A number of patients discussed complementary medicines including acupuncture (P9), therapeutic massage (P8, P25) and reflexology (P23). P2 talked at length about the reassurance, support and holistic care provided by the homeopath he had attended for several years, comparing interactions to those with their doctor: *'I used to get different doctors and you just felt as though you were part of a process but…my homeopath I go to, we sit and we talk about my symptoms, how I'm feeling'.*

Subtheme 5.3 Unmet emotional needs

Some patients highlighted unmet emotional support needs. P4, who was waiting for counselling, noted: '*I didn't feel like there was anybody professional shall I say, who I could talk to and just tell them how I felt and that I was really scared...'*. P25 commented that: *'[leaflets] could do [to include] a little bit more on your thoughts. Yeah on your mind, on the emotional side of it',* whilst P5 felt they would cope with their CHC psychologically, but '*at a price'.*

A number of patients identified key points in the CHC trajectory which caused emotional turmoil, but where they felt their needs were not met. Many highlighted the *'shock'* of diagnosis, which was described as *'a huge emotional rollercoaster'* (P7), while P13 talked about becoming upset when she discovered '*lumps'* which could indicate disease progression; others described feeling shock at being told they would need treatment after a period of observation (P3, P15). Despite the significance of these issues, patients identified a lack of emotional support at these critical junctures in their lives. Concerns could also be continually present, for example patients alluded to the difficulty of living with the uncertainty caused by their cancer diagnosis: '*it will flare up again...that causes anxiety… you don't know when…it's lurking there'* (P4).

DISCUSSION

This study provides novel in-depth insights about the experiences of CHC patients in seeking, accessing and engaging with psychosocial support. Many patients relied on support groups, which were generally perceived positively, but there were also significant barriers to their use, including: distress on hearing negative stories, lack of relevant groups due to the rarity of the cancer, and others' poor outcomes interrupting positive approaches to their illness. Family and friends provided psychosocial and practical support, though living with family did not guarantee this. Furthermore, individual patients' personality, outlook on life, and behaviour, impacted on their desire to seek and receive support, and on the family's ability to provide it, with some patients choosing not to disclose their diagnosis. Our study also indicated that positive interactions with HCPs, notably nursing staff, appeared to be largely determined by the patient's pathway; the majority of those who considered nurses as emotionally supportive had received treatment. Finally, both patients and relatives discussed the impact of the CHC on family and friends, stressing the needs of these groups and highlighting gaps in current provision.

For peer review only - http://bmjopen.bmj.com/site/about/guidelines.xhtml

The unique nature of CHCs likely impacts upon patients' experiences of psychosocial support. For example, patients who did not disclose their diagnosis tended to be those with the most indolent disease, who were being managed on observation, and/or who felt their cancer was not impacting their life and was invisible to others. This lack of disclosure may not have been possible for patients with cancers that are more visible (e.g. symptomatic and/or requiring immediate or aggressive treatment). Accordingly, some patients highlighted reluctance to help family and friends understand why treatment was not currently necessary, in contrast to expectations following a cancer diagnosis. In addition, unlike many potentially curable cancers, relapse and further intervention is always possible some CHCs, and for diagnoses, is expected. Therefore, the emotional impact of living with a CHC is likely to vary and fluctuate over time, particularly given patients' increasing life expectancy. Thus ongoing, yet potentially intermittent support, which is responsive to the patient's position on their relapsing-remitting pathway, and their associated needs, is especially important.

Our findings are consistent with previous research observing barriers to support group attendance, and thus opportunities for social support. For example, Swash et al (2018) describe patient difficulties in remaining positive when others with the same diagnosis become increasingly unwell, or die (18). A reliance on family and friends for social and emotional support has also been reported (26,27,37). Other research discusses patients withholding cancer related anxiety and distress from friends and relatives to protect them from worry (13). Generic (e.g. Macmillan) cancer support services were rarely mentioned by interviewees in our study and some deemed these inappropriate for CHC. This echoes previous research on CHCs, in which participants didn't always identify themselves as cancer patients, largely due to their prolonged pathway, lack of obvious symptoms, and associated terminology (e.g. haematology not oncology) (18). Limited HCP contact during observation has also been highlighted, with hospital appointments occurring at lengthy (e.g. 3-12 month) intervals, thus reducing opportunities for emotional support (18,23). Resonating with our study, some patients feel uncomfortable raising emotional concerns with HCPs, and feel HCPs do not want to discuss such issues, thereby enhancing barriers to support (18). The Serious Illness Care Program (SICP) may be a useful approach to improving communication between patients, families and HCPs (38). Initially designed as a palliative care intervention, recent research highlights the benefits of introducing SICP earlier in CHC patient journeys to encourage dialogue and enable discussion about fears and concerns associated with the cancer, beyond those related to end of life (39).

The recent National Cancer Patient Experience Survey (NCPES) found 92.8% of patients with blood cancer said they had a main contact, most often a nurse, to support them during treatment, with most finding these individuals reliable and helpful (40). HCPs are also in a position to recommend and increase awareness of sources of support (41), with the NCPES reporting that 89.1% of patients with blood cancers felt hospital staff provided guidance on support groups and other resources, and 78.4% indicating they received the right amount of support with their health and wellbeing. However, only 29.1% of those who felt they needed emotional support could access this at home post-treatment (40). This was slightly lower than the overall score for all cancer patients (31.8%), but is likely to be particularly relevant to those with CHCs, where there is no cure and thus further monitoring, and possibly treatment, is needed. Whilst clinical staff appreciate that CHC patients' emotions may change over time (42), they may underestimate the impact of these diseases on individual lives and psychological wellbeing (15,43), and research is required to fully understand patient needs at various timepoints. Furthermore, clinical staff, including CNSs, may lack the confidence, time and resources to deal with the psychosocial consequences of cancer (19,41), or may use their time to focus on physical disease aspects (44,45), which could result in the under provision of emotional support (17). Further research should consider the feasibility of HCPs

For peer review only - http://bmjopen.bmj.com/site/about/guidelines.xhtml

providing psychosocial care in clinical practice, and whether additional resources, including dedicated training, may be required.

Many sources of support discussed by patients are likely to have been impacted by the COVID-19 pandemic, due to lockdown restrictions and infection concerns, given that patients with haematological cancers are particularly vulnerable (46). This could result in some choosing to avoid face-to-face groups and services, or being encouraged to do so by HCPs. Online peer support was discussed by patients, and has been found to improve psychosocial wellbeing in cancer patients, but is recommended as complementary to face-to-face support rather than a replacement (47). Providing counselling or similar psychological support via videoconferencing has also been suggested, though barriers have been identified including: concerns regarding confidentiality, technical issues and software requirements (e.g. computer and webcam), and whether patients are able to talk openly in private, whilst in their own home (48,49).

Previous research has discussed the impact of cancer on patients' families and carers, including worsening psychological wellbeing, physical health and financial security (50–53), which may vary throughout illness (54). Suggestions to improve family outcomes include formally measuring caregiver needs, education and empowerment (55). A review of measures to assess the impact of caring found that few such tools had been systematically evaluated for cancer populations (56). There were also gaps, with a limited number covering changing family dynamics (which we found to be important), and many measures being outdated, thus potentially irrelevant, especially for people living longer with cancer (56). CHCs present specific problems due to their relapsing-remitting nature and the anxiety, repeated upheaval and on-going uncertainty for patients and families. The loneliness and confusion experienced by some family members in our study suggests that peer support would be useful. A recent feasibility study found one-to-one peer support was acceptable and beneficial for caregivers of those with newly diagnosed haematological cancers (57), with similar findings for group-based support (58).

This study has allowed us to capture a breadth of experiences and changing attitudes by interviewing patients at varying intervals post diagnosis, and with different treatment pathways. Although prolonged time since diagnosis may affect recall, referring back to diaries and calendars is likely to have mitigated this; moreover, in many cases, patient accounts were corroborated by relatives. Relatives were not present at every interview, a study limitation, especially with regard to views about support for carers and relatives. Our findings are likely to be transferable within the UK, and in countries with similar approaches to healthcare. However, we note that our sample only includes those over 50 years of age, and whilst the majority of CHCs are diagnosed in this population, the concerns of younger adults with CHCs may not have been identified. This should be considered in future research, as concerns among this group are likely to differ, possibly including greater emphasis on the potential impact of the CHC on fertility and family life (59), or the potential for symptoms and/or treatment side effects to impact patients' professional lives, either due to needing to retire or having to adapt their working patterns (37,60,61).

Conclusions

Individual preferences and attitudes resulted in marked differences concerning perceived needs for support, whether and how this was accessed, and the sources utilised. Relationships with others, including HCPs and family members, influenced whether support was considered to be available and if patients drew upon it. Preferences were also found to vary within individuals over time, mainly due to the long-term, changing nature of CHCs; the need for treatment after observation often leading patients to seek/receive additional support. Patients on observation may have specific

For peer review only - http://bmjopen.bmj.com/site/about/guidelines.xhtml

difficulties accessing psychosocial support, due to decisions about diagnostic disclosure and limited HCP interactions, which should be considered if such patients are not to be overlooked. Unmet needs were also evident, especially at key points of the clinical pathway, such as diagnosis and relapse, but also more generally, over time. Patients with CHCs may live with distress related to their condition for many years; thus, monitoring emotional needs is of equal importance as physical symptoms, particularly as the latter may never occur. HCPs are ideally placed for this role, as well as appropriate onward sign-posting/referral; although restricted time and resources may necessitate consideration of alternative approaches, and further research may be required to assess feasible possibilities. Our findings underscore the need for patients with CHC to receive support that encompasses their psychosocial needs, and considers their unique treatment pathways and individual lives.

Figure 1. Diagram of the impact of patients' individual approaches on identified themes

Acknowledgements

The authors are grateful to the patients and relatives who participated in interviews and shared their experiences with us so openly.

Competing interests

None declared.

Funding

This work was supported by Cancer Research UK [29685], the National Institute for Health Research [RP-PG-0613-2002], and Blood Cancer UK [15037]. No funders were involved in the study design, data collection, analysis, interpretation or reporting.

Ethics approval

Details concerning HMRN's configuration, methods and ethical approvals can be found elsewhere (30). For the present study, ethical approval was provided by the London, City and East Research Ethics Committee (REC:16/ LO/0740).

Patient consent for publication

Not applicable.

Data availability statement

The datasets analysed during the current study are not publicly available as participants did not provide permission for their entire interview transcripts to be published, and ethical approval was not sought for this. A document containing the qualitative themes, and example codes and quotes is available from the authors on reasonable request.

For peer review only - http://bmjopen.bmj.com/site/about/guidelines.xhtml

For peer review only

Author contributions

DH, ER, AS and RP designed the study. AS identified potential participants. DM recruited participants and conducted interviews. Transcripts were coded and analysed by RS with input from DM, DH and AH. RS wrote the first draft of the manuscript, which was revised by DH, DM, AH, ER, AS and RP, who also commented on clinical aspects of the study. All authors read and approved the final manuscript.

For peer review only

For peer review only - http://bmjopen.bmj.com/site/about/guidelines.xhtml

REFERENCES

1. Swerdlow S, Campo E, Harris N, Jaffe E, Pileri S, Stein H, et al. WHO Classification of Tumours of Haematopoietic and Lymphoid Tissues. Lyon, France: World Health Organisation; 2017.

2. Sung H, Ferlay J, Siegel RL, Laversanne M, Soerjomataram I, Jemal A, et al. Global Cancer Statistics 2020: GLOBOCAN Estimates of Incidence and Mortality Worldwide for 36 Cancers in 185 Countries. CA: A Cancer Journal for Clinicians. 2021;71:209–49.

3. Office for National Statistics. Cancer registration statistics, England, 2017. Available from: https://www.ons.gov.uk/peoplepopulationandcommunity/healthandsocialcare/conditionsanddiseases/datasets/cancerregistrationstatisticscancerregistrationstatisticsengland (Accessed: June 2023)

4. Haematological Malignancy Research Network. Statistics. 2022. Available from: https://hmrn.org/statistics/incidence (Accessed: June 2023)

5. Howell DA, McCaughan D, Smith AG, Patmore R, Roman E. Incurable but treatable: Understanding, uncertainty and impact in chronic blood cancers—A qualitative study from the UK's Haematological Malignancy Research Network. PLOS ONE. 2022;10;17(2):e0263672.

6. Blood Cancer UK. Watch and wait. 2022. Available from: https://bloodcancer.org.uk/understanding-blood-cancer/watch-wait/ (Accessed: June 2023)

7. Bewarder M, Stilgenbauer S, Thurner L, Kaddu-Mulindwa D. Current Treatment Options in CLL. Cancers. 2021;13(10):2468.

8. Matasar MJ, Luminari S, Barr PM, Barta SK, Danilov AV, Hill BT, et al. Follicular Lymphoma: Recent and Emerging Therapies, Treatment Strategies, and Remaining Unmet Needs. Oncologist. 2019;24(11):e1236–50.

9. Rajkumar SV. Multiple myeloma: Every year a new standard? Hematol Oncol. 2019;37 Suppl 1:62–5.

10. Allart-Vorelli P, Porro B, Baguet F, Michel A, Cousson-Gélie F. Haematological cancer and quality of life: a systematic literature review. Blood Cancer Journal. 2015;5(4):e305.

11. Goswami P, Oliva EN, Ionova T, Else R, Kell J, Fielding AK, et al. Quality-of-life issues and symptoms reported by patients living with haematological malignancy: a qualitative study. Therapeutic Advances in Hematology. 2020;11:2040620720955002.

12. Waweru C, Kaur S, Sharma S, Mishra N. Health-related quality of life and economic burden of chronic lymphocytic leukemia in the era of novel targeted agents. Current Medical Research and Opinion. 2020;36(9):1481–95.

13. Maher K, Vries KD. An exploration of the lived experiences of individuals with relapsed Multiple Myeloma. European Journal of Cancer Care. 2011;20(2):267–75.

For peer review only - http://bmjopen.bmj.com/site/about/guidelines.xhtml

14. Molassiotis A, Wilson B, Blair S, Howe T, Cavet J. Unmet supportive care needs, psychological well-being and quality of life in patients living with multiple myeloma and their partners. Psychooncology. 2011;20(1):88–97.

15. Evans J, Ziebland S, Pettitt AR. Incurable, invisible and inconclusive: watchful waiting for chronic lymphocytic leukaemia and implications for doctor-patient communication. European Journal of Cancer Care. 2012;21(1):67–77.

16. Lamers J, Hartmann M, Goldschmidt H, Brechtel A, Hillengass J, Herzog W. Psychosocial support in patients with multiple myeloma at time of diagnosis: who wants what? Psycho-Oncology. 2013;22:2313-2320

17. Swash B, Hulbert-Williams N, Bramwell R. Unmet psychosocial needs in haematological cancer: a systematic review. Supportive Care in Cancer. 2014;22(4):1131–41.

18. Swash B, Hulbert-Williams N, Bramwell R. ‘Haematological cancers, they’re a funny bunch’: A qualitative study of non-Hodgkin’s lymphoma patient experiences of unmet supportive care needs. Journal of Health Psychology. 2018;28;1464–75.

19. Hulin C, Hansen T, Heron L, Pughe R, Streetly M, Plate A, et al. Living with the burden of relapse in multiple myeloma from the patient and physician perspective. Leukemia Research. 2017;59:75–84.

20. Tsatsou I, Konstantinidis T, Kalemikerakis I, Adamakidou T, Vlachou E, Govina O. Unmet Supportive Care Needs of Patients with Hematological Malignancies: A Systematic Review. Asia Pacific Journal of Oncology Nursing. 2020;8(1):5–17.

21. LeBlanc MR, LeBlanc TW, Bryant AL, Pollak KI, Bailey DE, Smith SK. A qualitative study of the experiences of living my multiple myeloma. Oncology Nursing Forum. 2021;48(2):151–60.

22. Rubins Z, Gibson BJ, Chantry A. What Can Patient Narratives Reveal to Us About the Experience of a Diagnosis of Myeloma? A Qualitative Scoping Review. Journal of Patient Experience. 2022;9:23743735221079132.

23. Damen MDC, Westerweel PE, Levin MD, Pelle AJ. Unmet supportive care needs, anxiety and depression in haematology patients during watch-and-wait. Psycho-Oncology. 2022;31(2):176–84.

24. El-Jawahri AR, Vandusen HB, Traeger LN, Fishbein JN, Keenan T, Gallagher ER, et al. Quality of life and mood predict posttraumatic stress disorder after hematopoietic stem cell transplantation. Cancer. 2016;122(5):806–12.

25. Haematological Malignancy Research Network. Survival. 2022. Available from: https://hmrn.org/statistics/survival (Accessed: June 2023)

26. Cuffe CH, Quirke MB, McCabe C. Patients’ experiences of living with multiple myeloma. British Journal of Nursing. 2020;29(2):103–10.

27. Raphael D, Frey R, Gott M. Maintaining psychosocial wellbeing for post-treatment haematological cancer survivors: Strategies and potential barriers. European Journal of Oncology Nursing. 2019;38:36–41.

For peer review only

For peer review only - http://bmjopen.bmj.com/site/about/guidelines.xhtml

28. Tong A, Sainsbury P, Craig J. Consolidated criteria for reporting qualitative research (COREQ): a 32-item checklist for interviews and focus groups. International Journal for Quality in Health Care. 2007;19(6):349–57.

29. Haematological Malignancy Research Network. 2022. Available from: https://www.hmrn.org (Accessed: June 2023)

30. Roman E, Kane E, Howell D, Lamb M, Bagguley T, Crouch S, et al. Cohort Profile Update: The Haematological Malignancy Research Network (HMRN)'s UK population-based cohorts. International Journal of Epidemiology. 2022;e87–94.

31. Patton MQ. Qualitative Research and Evaluation Methods: Integrating Theory and Practice. Fourth Edition. Thousand Oaks, CA: Sage Publications; 2015.

32. Bowen GA. Naturalistic inquiry and the saturation concept: a research note. Qualitative Research. 2008;8(1):137–52.

33. Atherton K, Young B, Salmon P. Understanding the information needs of people with haematological cancers. A meta-ethnography of quantitative and qualitative research. European Journal of Cancer Care. 2017;26(6).

34. Atherton K, Young B, Kalakonda N, Salmon P. Perspectives of patients with haematological cancer on how clinicians meet their information needs: 'managing' information versus 'giving' it. Psychooncology. 2018;27(7):1719–26.

35. Braun V, Clarke V. One size fits all? What counts as quality practice in (reflexive) thematic analysis? Qualitative Research in Psychology. 2021;18(3):328–52.

36. Braun V, Clarke V. Conceptual and design thinking for thematic analysis. Qualitative Psychology. 2022;9(1):3–26.

37. Molassiotis A, Wilson B, Blair S, Howe T, Cavet J. Living with multiple myeloma: experiences of patients and their informal caregivers. Supportive Care in Cancer. 2011;19(1):101–11.

38. Bernacki R, Hutchings M, Vick J, Smith G, Paladino J, Lipsitz S, et al. Development of the Serious Illness Care Program: a randomised controlled trial of a palliative care communication intervention. BMJ Open. 2015;5(10):e009032.

39. Borregaard Myrhøj C, Novrup Clemmensen S, Sax Røgind S, Jarden M, Toudal Viftrup D. Serious illness conversations in patients with multiple myeloma and their family caregivers—A qualitative interview study. European Journal of Cancer Care. 2022;31(1):e13537.

40. NHS England. National Cancer Patient Experience Survey. 2021. Available from: https://www.ncpes.co.uk/2021-national-results/ (Accessed: June 2023)

41. Newton JC, O'Connor M, Saunders C, Moorin R, Ali S, Nowak AK, et al. The role of psychosocial support in the experiences of people living with advanced cancer: A qualitative exploration of patients' perspectives. Psycho-Oncology. 2021;30(3):287–95.

42. Liptrott SJ, Bee P, Lovell K. Providing Telephone-Based Support for Patients With Haematological Malignancies: A Qualitative Investigation of Expert Nurses. Canadian Journal of Nursing Research. 2022;54(2):121–33.

For peer review only - http://bmjopen.bmj.com/site/about/guidelines.xhtml

43. Hewison A, Roman E, Smith A, McCaughan D, Sheridan R, Patmore R, et al. Chronic myeloid leukemia: A qualitative interview study exploring disease impact from patient and practitioner perspectives. In Review. Available from: https://www.researchsquare.com/article/rs-2065284/v1 (Accessed: June 2023)

44. NHS National Cancer Programme. Excellence in Cancer Care: The Contribution of the Clinical Nurse Specialist. 2010. Available from: https://www.macmillan.org.uk/documents/aboutus/commissioners/excellenceincancercarethecontributionoftheclinicalnursespecialist.pdf (Accessed: June 2023)

45. Independent Cancer Taskforce. Achieving World-Class Cancer Outcomes - A Strategy for England 2015-2020. 2015. Available from: https://cancervanguard.nhs.uk/national-cancer-strategy/ (Accessed: June 2023)

46. Zomerdijk N, Jongenelis M, Yuen E, Turner J, Huntley K, Smith A, et al. Experiences and needs of people with haematological cancers during the COVID-19 pandemic: A qualitative study. Psycho-Oncology. 2022;31(3):416–24.

47. Allison KR, Patterson P, Guilbert D, Noke M, Husson O. Logging On, Reaching Out, and Getting By: A Review of Self-reported Psychosocial Impacts of Online Peer Support for People Impacted by Cancer. Proceedings of the ACM on Human-Computer Interaction. 2021;5(CSCW1):95:1-95:35.

48. Siegel A, Zuo Y, Moghaddamcharkari N, McIntyre RS, Rosenblat JD. Barriers, benefits and interventions for improving the delivery of telemental health services during the coronavirus disease 2019 pandemic: a systematic review. Current Opinion in Psychiatry. 2021;34(4):434–43.

49. Connolly SL, Miller CJ, Lindsay JA, Bauer MS. A systematic review of providers' attitudes toward telemental health via videoconferencing. Clinical Psychology: Science and Practice. 2020;27(2):e12311.

50. Girgis A, Lambert S, Johnson C, Waller A, Currow D. Physical, Psychosocial, Relationship, and Economic Burden of Caring for People With Cancer: A Review. Journal of Oncology Practice. 2013;9(4):197–202.

51. Pitceathly C, Maguire P. The psychological impact of cancer on patients' partners and other key relatives: a review. European Journal of Cancer. 2003;39(11):1517–24.

52. Williams AL. Psychosocial burden of family caregivers to adults with cancer. Psycho-Oncology. 2013;73–85.

53. Geng HM, Chuang DM, Yang F, Yang Y, Liu WM, Liu LH, et al. Prevalence and determinants of depression in caregivers of cancer patients. Medicine. 2018;97(39):e11863.

54. Kim Y, Given BA. Quality of life of family caregivers of cancer survivors. Cancer. 2008;112(S11):2556–68.

55. Berry LL, Dalwadi SM, Jacobson JO. Supporting the supporters: What family caregivers need to care for a loved one with cancer. Journal of Oncology Practice. 2016;13(1):8.

56. Shilling V, Matthews L, Jenkins V, Fallowfield L. Patient-reported outcome measures for cancer caregivers: a systematic review. Quality of Life Research. 2016;25(8):1859–76.

For peer review only - http://bmjopen.bmj.com/site/about/guidelines.xhtml

57. Husted Nielsen I, Piil K, Tolver A, Grønbæk K, Kjeldsen L, Jarden M. Family caregiver ambassador support for caregivers of patients with newly diagnosed hematological cancer: a feasibility study. Supportive Care in Cancer. 2022;30(8):6923–35.

58. Pailler ME, Johnson TM, Zevon MA, Kuszczak S, Griffiths E, Thompson J, et al. Acceptability, Feasibility, and Efficacy of a Supportive Group Intervention for Caregivers of Newly Diagnosed Leukemia Patients. Journal of Psychosocial Oncology. 2015;33(2):163–77.

59. Devine H, Verina D. Young Adults with Multiple Myeloma. Seminars in Oncology Nursing. 2017;33(3):316–31.

60. Potrata B, Cavet J, Blair S, Howe T, Molassiotis A. 'Like a sieve': an exploratory study on cognitive impairments in patients with multiple myeloma. European Journal of Cancer Care. 2010;19(6):721–8.

61. Forbat L, Sedgley T, Bellussi L. Multiple Myeloma in People of Working Age: A Systematic Review and Evidence Synthesis of Experiences of Paid and Unpaid Work. European Journal of Cancer Care. 2023;e6292479.

For peer review only

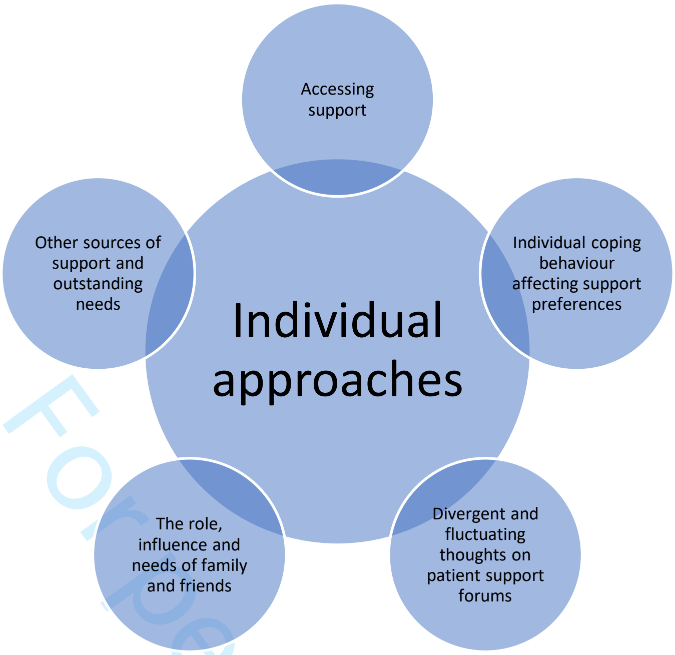

Figure 1. Diagram of the impact of patients' individual approaches on identified themes

For peer review only

Supplementary file 1
Details of sample

ID	Diagnosis[1]	Age range at interview (years)	Years since diagnosis	Known treatment line(s) preceeding interview[2,3]					
				1	2	3	4	5	6
P1	CLL	60-70	4	Observation	-	-	-	-	-
P2	MZL	60-70	15	Observation	Chemotx	Observation	-	-	-
P3	CLL	60-70	22	Observation	Chemotx	Observation	-	-	-
P4	MZL	60-70	3	Observation	Chemotx	-	-	-	-
P5	MZL	50-60	2	HPE	Observation	-	-	-	-
P6*	CLL	70-80	8	Observation	Chemotx	Observation	-	-	-
P7*	CLL	60-70	6	Observation	Chemotx	Observation	-	-	-
P8	FL	70-80	3	Chemotx	Radiotx	Observation	-	-	-
P9	CLL	80-90	5	Observation	Chemotx	-	-	-	-
P10	FL	70-80	8	Observation	Chemotx	Chemotx	Chemotx	-	-
P11	Myeloma	60-70	10	Observation	Chemotx	Observation	-	-	-
P12	MZL	70-80	5	Observation	Chemotx	-	-	-	-
P13	CLL	50-60	1	Observation	-	-	-	-	-
P14	Myeloma	60-70	4	Steroids	Radiotx	Chemotx	Chemotx	Chemotx	SCT
P15	FL	70-80	3	Observation	Chemotx	-	-	-	-
P16	Myeloma	60-70	2	Chemotx	Chemotx	Chemotx	SCT	Observation	-
P17*	FL	60-70	3	Observation	-	-	-	-	-
P18	Myeloma	60-70	3	Chemotx	Chemotx	Chemotx	SCT	Observation	
P19	FL	50-60	3	Steroids	Chemotx	Chemotx	Observation	-	-
P20*	CLL	70-80	4	Observation	-	-	-	-	-
P21*	Myeloma	70-80	3	Steroids	Chemotx	Chemotx	Chemotx	SCT	-
P22*	CLL	70-80	3	Observation	Clinical trial	Observation	-	-	-
P23	Myeloma	60-70	3	Observation	-	-	-	-	-
P24	FL	50-60	4	Steroids	Chemotx	Radiotx	Observation	-	-
P25	FL	60-70	4	Chemotx	Chemotx	-	-	-	-
P26	Myeloma	70-80	4	Observation	-	-	-	-	-
P27*	CLL	70-80	4	Chemotx	Observation	-	-	-	-
P28	Myeloma	60-70	4	Steroids	Chemotx	Chemotx	SCT	Clinical trial	Chemotx
P29	CLL	70-80	3	Clinical trial	Observation	-	-	-	-
P30*	Myeloma	70-80	2	Observation	-	-	-	-	-
P31*	Myeloma	70-80	2	Radiotx	Steroids	Chemotx	Observation	-	-
P32*	MZL	60-70	2	Observation	Chemotx	Observation	-	-	-
P33	Myeloma	50-60	3	Chemotx	Chemotx	SCH	Observation	-	-
P34	FL	50-60	4	Steroids	Chemotx	Chemotx	Chemotx	-	-
P35	Myeloma	50-60	2	Chemotx	Chemotx	Chemotx	Chemotx	SCT	Observation

[1] CLL – Chronic Lymphocytic Leukaemia; FL – Follicular Lymphoma; MZL – Systemic Marginal Zone Lymphoma.

[2] Chemotx = Chemotherapy; HPE = H. Pylori eradication; Radiotx = Radiotherapy; SCT = Stem cell transplant (all autografts); SCH = Stem cell harvest (shown as SCT did not take place).

[3] Does not include supportive care (e.g. blood product transfusions, plasma exchange, bisphosphonates, cell mobilization products)

*Relative present at interview

For peer review only

Supplementary file 2

Topic guide

(Tested with two patients prior to use in interviews)

Patients and relatives were asked to focus on key time-points (diagnosis, W&W, treatment), with respect to the topics included in the following three areas:

1) Understanding and impact of chronic blood cancer

- Looking back, what did you know about blood cancers, and your diagnosis specifically?
- What led you to your diagnosis initially? *(symptoms, suspected cancer?)*
- How important is it to you that you receive information about your cancer? *(why is that?)*
- How do you feel about the information given to you at diagnosis/start of treatment?
- What did you understand about your diagnosis? *(and others?)*
- What were your expectations of what would happen after diagnosis? *(treatment, follow-up)*
- What issues did you discuss at the time of your diagnosis? *(disease type, likely pathway, outcomes?)*
- What did you understand about your likely pathway and outcomes? *(immediate and later?)*
- What do you understand about your treatment?
- How do you feel about the future? *(optimistic, pessimistic?)*
- What impact has having a chronic blood cancer had on you? (activities, work, psychosocial issues)
- What strategies have you used to help you cope with your blood cancer?

2) Information

- How do you feel about getting information from HCPs generally? *(time constraints; use of language/terminology)*
- Do you feel the information given applies specifically to you? *(personalized, tailored, specific)*
- How healthcare practitioners (HCPs) ascertain your information needs?
- Is the information you received explained in a way you can understand? *(technical language; level of detail)*
- What do HCPs do to check if you understand the information they give you?
- How do you feel about asking questions? Are your questions always answered?
- Do you feel that your information needs are usually met? What worked well and could have been better? *(diagnosis; treatment initiation/cessation - examples)*
- What do you think about the timing of information from HCPs? When is the right time? *(at diagnosis; during clinic appointments; when disease status changes; at other times)*
- How do/did you feel about discussing the risks/benefits of different treatments with HCPs?
- How do you feel about discussing prognosis? *("a statement about expectations that refers to the likely course of the cancer and/or outcome") (want to know/not; timing; language)*
- What strategies do you use to absorb information? (*in general, how bad news is processed*)

3) Treatment decisions

- How do you feel about being involved in decisions with HCPs about your treatment?
- Have you been asked you if you want to be involved in decisions about treatment?
- Do you want to be involved in decisions? *(preference for patient only; clinician only; patient/clinician)*
- What should be considered during treatment decision making? *(effectiveness of treatment; side effects; prognosis; patient goals, values, preferences; impact on quality of life)*
- What might make it easier or harder for you to be involved in making decisions about your treatment? *(time; style of communication; how information is conveyed; explanations)*
- Are there particular time-points when it is harder to be involved in making decisions about treatment? *(diagnosis; treatment initiation/change; treatment cessation)*

For peer review only

COREQ (COnsolidated criteria for REporting Qualitative research) Checklist

No. Item	Guide questions/description	Page	Further detail
**Domain 1: Research team and reflexivity**			
**Personal characteristics**			
1. Inter viewer/facilitator	Which author/s conducted the interview or focus group?	5	DM
2. Credentials	What were the researcher's credentials? E.g. PhD, MD	n/a	BA, RN, MSc
3. Occupation	What was their occupation at the time of the study?	n/a	Senior Research Fellow
4. Gender	Was the researcher male or female?	5	Female
5. Experience and training	What experience or training did the researcher have?	n/a	Registered Nurse, conducted many qualitative interviews.
**Relationship with participants**			
6. Relationship established	Was a relationship established prior to study commencement?	n/a	No
7. Participant knowledge of the interviewer	What did the participants know about the researcher? e.g. personal goals, reasons for doing the research	n/a	No knowledge.
8. Interviewer characteristics	What characteristics were reported about the interviewer/facilitator? e.g. Bias, assumptions, reasons and interests in the research topic	n/a	Experienced qualitative health services researcher.
**Domain 2: Study design**			
**Theoretical framework**			
9. Methodological orientation and Theory	What methodological orientation was stated to underpin the study? e.g. grounded theory, discourse analysis, ethnography, phenomenology, content analysis	5	Reflexive thematic analysis.
**Participant selection**			
10. Sampling	How were participants selected? e.g. purposive, convenience, consecutive, snowball	5	Purposive.
11. Method of approach	How were participants approached? e.g. face-to-face, telephone, mail, email	5	Mail, followed up by telephone if expressed interest.
12. Sample size	How many participants were in the study?	5	35
13. Non-participation	How many people refused to participate or dropped out? Reasons?	n/a	None dropped out. 20 patients did not respond to invite for reasons unknown.
**Setting**			
14. Setting of data collection	Where was the data collected? e.g. home, clinic, workplace	5	Home, University.
15. Presence of non-participants	Was anyone else present besides the participants and researchers?	5	Relatives were present at 10 interviews.
16. Description of sample	What are the important characteristics of the sample? e.g. demographic data, date	5	Also found in Supplementary file 2.
**Data collection**			

For peer review only

17. Interview guide	Were questions, prompts, guides provided by the authors? Was it pilot tested?	5	Topic guide found in Supplementary file 1.
18. Repeat interviews	Were repeat inter views carried out? If yes, how many?	n/a	No repeat interviews were carried out.
19. Audio/visual recording	Did the research use audio or visual recording to collect the data?	5	Audio.
20. Field notes	Were field notes made during and/or after the interview or focus group?	n/a	No.
21. Duration	What was the duration of the inter views or focus group?	5	60-90 minutes
22. Data saturation	Was data saturation discussed?	n/a	Yes, interviews were continued until saturation.
23. Transcripts returned	Were transcripts returned to participants for comment and/or correction?	n/a	No transcripts were returned.
**Data analysis**			
24. Number of data coders	How many data coders coded the data?	5	RS, DM, DH, AH
25. Description of the coding tree	Did authors provide a description of the coding tree?	Figure 1	
26. Derivation of themes	Were themes identified in advance or derived from the data	F5	Data manged in Microsoft Word.
27. Software	What software, if applicable, was used to manage the data	n/a	No software was used.
28. Participant checking	Did participants provide feedback on findings?	n/a	No.
**Reporting**			
29. Quotations presented	Were participant quotations presented to illustrate the themes/findings? Was each quotation identified? e.g. participant number	6-11	Quotations identified by participant number.
30. Data and findings consistent	Was there consistency between the data presented and the findings?	6-11	Yes.
31. Clarity of major themes	Were major themes clearly presented in the findings?	6-11, Figure 1	Major themes were presented alongside subthemes.
32. Clarity of minor themes	Is there a description of diverse cases or discussion of minor themes?	6-11	Yes, a range of responses are considered.

Developed from: Tong A, Sainsbury P, Craig J. Consolidated criteria for reporting qualitative research (COREQ): a 32-item checklist for interviews and focus groups. *International Journal for Quality in Health Care*. 2007. Volume 19, Number 6: pp. 349 – 357

For peer review only
